# Suppression of abscisic acid biosynthesis at the early infection stage of *Verticillium longisporum* in oilseed rape (*Brassica napus*)

**DOI:** 10.1111/mpp.12867

**Published:** 2019-10-11

**Authors:** Falk H. Behrens, Dirk Schenke, Roxana Hossain, Wanzhi Ye, Markus Schemmel, Thomas Bergmann, Claudia Häder, Yan Zhao, Lena Ladewig, Wenxuan Zhu, Daguang Cai

**Affiliations:** ^1^ Institute of Phytopathology Department of Molecular Phytopathology and Biotechnology Christian‐Albrechts‐University of Kiel Hermann Rodewald Str. 9 D‐24118 Kiel Germany

**Keywords:** ABA, *Brassica napus*, NCED3, RNA-Seq, *Verticillium longisporum*

## Abstract

*Verticillium longisporum* infects oilseed rape (*Brassica napus*) and *Arabidopsis thaliana*. To investigate the early response of oilseed rape to the fungal infection, we determined transcriptomic changes in oilseed rape roots at 6 days post‐inoculation (dpi) by RNA‐Seq analysis, in which non‐infected roots served as a control. Strikingly, a subset of genes involved in abscisic acid (ABA) biosynthesis was found to be down‐regulated and the ABA level was accordingly attenuated in 6 dpi oilseed rape as compared with the control. Gene expression analysis revealed that this was mainly attributed to the suppression of *BnNCED3*‐mediated ABA biosynthesis, involving, for example, *BnWRKY57*. However, this down‐regulation of ABA biosynthesis could not be observed in infected *Arabidopsis* roots. *Arabidopsis* ABA‐ defective mutants *nced3* and *aao3* displayed pronounced tolerance to the fungal infection with delayed and impeded symptom development, even though fungal colonization was not affected in both mutants. These data suggest that ABA appears to be required for full susceptibility of *Arabidopsis* to the fungal infection. Furthermore, we found that in both 6 dpi oilseed rape and the Arabidopsis *nced3* mutant, the salicylic acid (SA) signalling pathway was induced while the jasmonic acid (JA)/ethylene (ET) signalling pathway was concomitantly mitigated. Following these data, we conclude that in oilseed rape the *V. longisporum* infection triggers a host‐specific suppression of the NCED3‐mediated ABA biosynthesis, consequently increasing plant tolerance to the fungal infection. We believe that this might be part of the virulence strategy of *V. longisporum* to initiate/establish a long‐lasting compatible interaction with oilseed rape (coexistence), which appears to be different from the infection process in *Arabidopsis*.

## Introduction

*Brassica napus* (oilseed rape) is an important oilseed crop grown worldwide and the cultivated area of oilseed rape has continuously increased in recent years (FAOSTAT, 2016). Lack of sufficient crop rotation and tillage operations results in an enrichment of soilborne pathogens, posing a real threat to crop cultivation (De Coninck *et al.*, 2015). *Verticillium longisporum* is a hemibiotrophic soilborne pathogen causing verticillium stem striping in susceptible oilseed rape (De Coninck *et al.*, 2015; Depotter *et al.*, 2016). In this compatible interaction fungal hyphae enter the host through the roots without developing specific infection structures, and grow intra‐ and intercellularly through the root cortex towards the central cylinder. Fungal colonization of xylem vessels was thought to affect the xylem function and the transpiration stream, restricting water and nutrient transportation (Depotter *et al.*, 2016; Eynck *et al.*, 2007; Floerl *et al.*, 2008), but a recent study showed that *V. longisporum* does not reduce xylem function in *Brassica napus* (Lopisso *et al.*, 2017). In the field, symptoms on winter oilseed rape plants, including stem striping, appear in the late growing season, whereas stunted growth, leaf chlorosis as well as reduced leaf area are typically observable on root dip‐inoculated younger plants (Depotter *et al.*, 2016; Eynck *et al.*, 2009). This, together with the fungal growth within the plant, prevents an efficient disease management (Eynck *et al.*, 2007; Happstadius *et al.*, 2003). Efforts have been made to introduce *V. longisporum* resistance from *B. rapa* and *B. oleracea* genomes via resynthesis approaches, and several genotypes with increased resistance have been reported (Happstadius *et al.*, 2003; Rygulla *et al.*, 2007a, 2007b). However, so far no highly resistant germplasm of *B. napus* is available for cultivation.

Intriguingly, this plant–fungus interaction appears to involve post‐transcriptional gene silencing (PTGS) (Ellendorff *et al.*, 2009). Argonaute (AGO) proteins and microRNAs directly modulate the infection process of *Verticillium* fungi and there is increasing evidence that noncoding RNAs play a pivotal role in cross‐kingdom RNA interference (Shen *et al.*, 2014; Wang *et al.*, 2016; Zhang *et al.*, 2016). On the phytohormone level the plant–*V. longisporum* interaction has also been investigated in *Arabidopsis thaliana* (Floerl *et al.*, 2012; Iven *et al.*, 2012; Johansson *et al.*, 2006; Ralhan *et al.*, 2012), particularly regarding the involvement and role of phytohormone pathways relying on salicylic acid (SA) and jasmonic acid (JA) derivatives or ethylene (ET). It has been demonstrated that *V. longisporum* requires a *COI1*‐dependent but JA‐Ile‐independent mechanism to efficiently colonize *Arabidopsis* (Ralhan *et al.*, 2012). However, since the Arabidopsis *npr1‐1* mutant is more susceptible and shows decreased responses to the ethylene precursor 1‐aminocyclopropane‐1‐carboxylic acid (ACC) or methyl jasmonate (MeJA) pretreatments, involvement of the SA pathway in the plant–fungus interaction was also postulated (Johansson *et al.*, 2006). Another indication for the involvement of ET and SA in the *Arabidopsis*–*V. longisporum* interaction came from the observation that SA‐dependent *PR1* and *PR2* as well as the ET‐dependent *PR4* expression was elevated at 7 days post‐inoculation (dpi) (Johansson *et al.*, 2006).

The role of abscisic acid (ABA) in plant–pathogen interactions is somewhat controversial. ABA is generally believed to benefit plant resistance to abiotic stress, but it is also involved in stomatal closure and callose production and can therefore contribute to resistance against bacteria (Ton *et al.*, 2009). An increased permeability of the cuticle and enhanced reactive oxygen species (ROS) production were observed in *aba2* and *aba3* mutants, which resulted in increased resistance to *Botrytis cinerea* (Cui *et al.*, 2016; L’Haridon *et al*., 2011). ABA can contribute to plant defence against necrotrophic pathogens via enhancement of the JA response (Lorenzo *et al.*, 2004). Hok *et al. *(2014) reported that the *Arabidopsis* ABA signalling mutant *abi1‐1* was more susceptible to *Hyaloperonospora arabidopsidis* (*Hpa*), which is different from the observations made for *V. longisporum* by Johansson *et al. *(2006). Elevated ABA levels were found to be correlated with increased rice susceptibility to *Xanthomonas oryzae* pv. *oryzae* (Xu *et al.*, 2013) and ABA was reported to negatively regulate the elicitor‐induced synthesis of the phytoalexin capsidiol in tobacco (Mialoundama *et al.*, 2009) or to abolish the PTI‐induced chloroplastic reactive oxidative burst (de Torres‐Zabala *et al*., 2015) as well. Some bacterial pathogens are even able to manipulate the phytohormone homeostasis by increasing ABA levels in plants, thereby compromising plant defence reactions (de Torres‐Zabala *et al.*, 2007, 2009). As demonstrated in the *Ustilago maydis*–*Zea mays* pathosystem, the fungus was capable of producing ABA, resulting in increased ABA levels within the plant cells at the early infection stages, which probably serves as part of the fungal virulence strategy (Morrison *et al.*, 2015). Similar observations have been reported by works on *B. cinerea, Cercospora* spp., *Fusarium* spp., *Rhizoctonia* spp., *Magnaporthe grisea* and *Magnaporthe oryzae* (Cao *et al.*, 2011; Siewers *et al.*, 2006; Spence *et al.*, 2015). On the other hand, ABA accumulation was suppressed during infection of *Arabidopsis* with *Alternaria brassicicola* (Flors *et al.*, 2008). ABA promoted plant susceptibility to *B. cinerea*, while contributing to resistance against *Sclerotinia sclerotiorum* (Mbengue *et al.*, 2016). An increase in SA was reported in xylem sap of oilseed rape after infection with *V.* *longisporum* (Ratzinger *et al.*, 2009). Thus, depending on the plant–pathogen system, ABA can influence plant defence response in a positive or negative manner. Nevertheless, the role of ABA in the *B. napus*–*V. longisporum* interaction remains largely unsolved.

Although molecular understanding of plant–*Verticillium* interactions has been achieved with the model plant *A. thaliana* in recent years, less data is available for oilseed rape. The recently published genome sequence of *B. napus* (Chalhoub *et al.*, 2014; Sun *et al.*, 2017) and advanced parallel deep‐sequencing technology provide an opportunity to globally investigate the reprogramming of gene expression of oilseed rape in response to pathogen attacks. To get a deeper insight into the early transcriptional responses of oilseed rape to *Verticillium* infection, we conducted a comparative transcriptomic analysis between *Verticillium*‐infected and non‐infected oilseed rape at 6 dpi using RNA‐Seq experiments. Interestingly, a large fraction of ABA biosynthesis‐related genes was found to be significantly suppressed in the oilseed rape roots after infection with *V. longisporum* isolate 43 (*Vl43*), but not in *Arabidopsis* roots. This provoked us to hypothesize a possible role of ABA suppression in the early oilseed rape–fungus interaction, being distinct from *Arabidopsis*. Here, we report that the *Vl43* infection triggers suppression of *NCED3*‐mediated ABA biosynthesis in the oilseed rape roots at the early infection stage. We demonstrate that ABA is required for full susceptibility of *Arabidopsis* to *Vl43* infection. Thus, in oilseed rape the fungus‐induced suppression of ABA at the early infection stage may be part of the virulence mechanism, allowing the fungus to establish a long‐lasting compatible interaction with oilseed rape, which differs from the *Arabidopsis*–*V. longisporum* pathosystem.

## Results

### Transcriptomic analysis indicates suppression of ABA‐related gene expression in oilseed rape roots at the early infection stage

**Figure 1:**
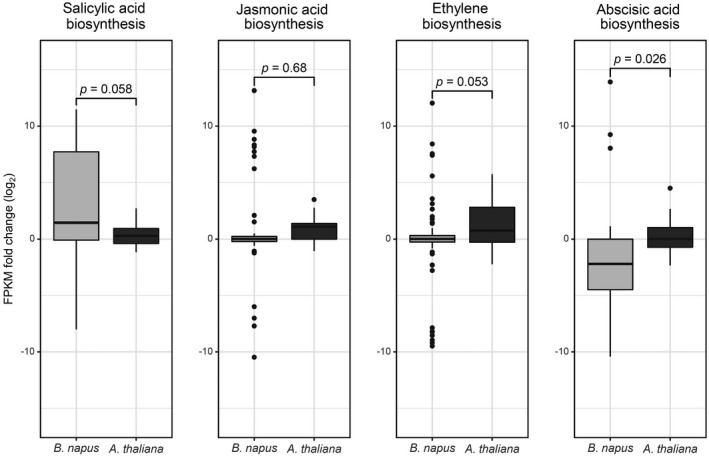
*Verticillium longisporum‐*induced differential expression of genes annotated to stress‐related phytohormone biosynthetic processes. For both plant species boxplots represent the log_2_ fold change expression of all genes annotated to the GO terms for salicylic acid (GO:0009697), jasmonic acid (GO:0009695), ethylene (GO:0009693) and abscisic acid (GO:0009688) biosynthetic processes that were differentially regulated in at least one species. *P*‐values calculated by Welch *t*‐tests indicate the significant difference between *Brassica napus* and *Arabidopsis thaliana* for each process.

**Figure 2:**
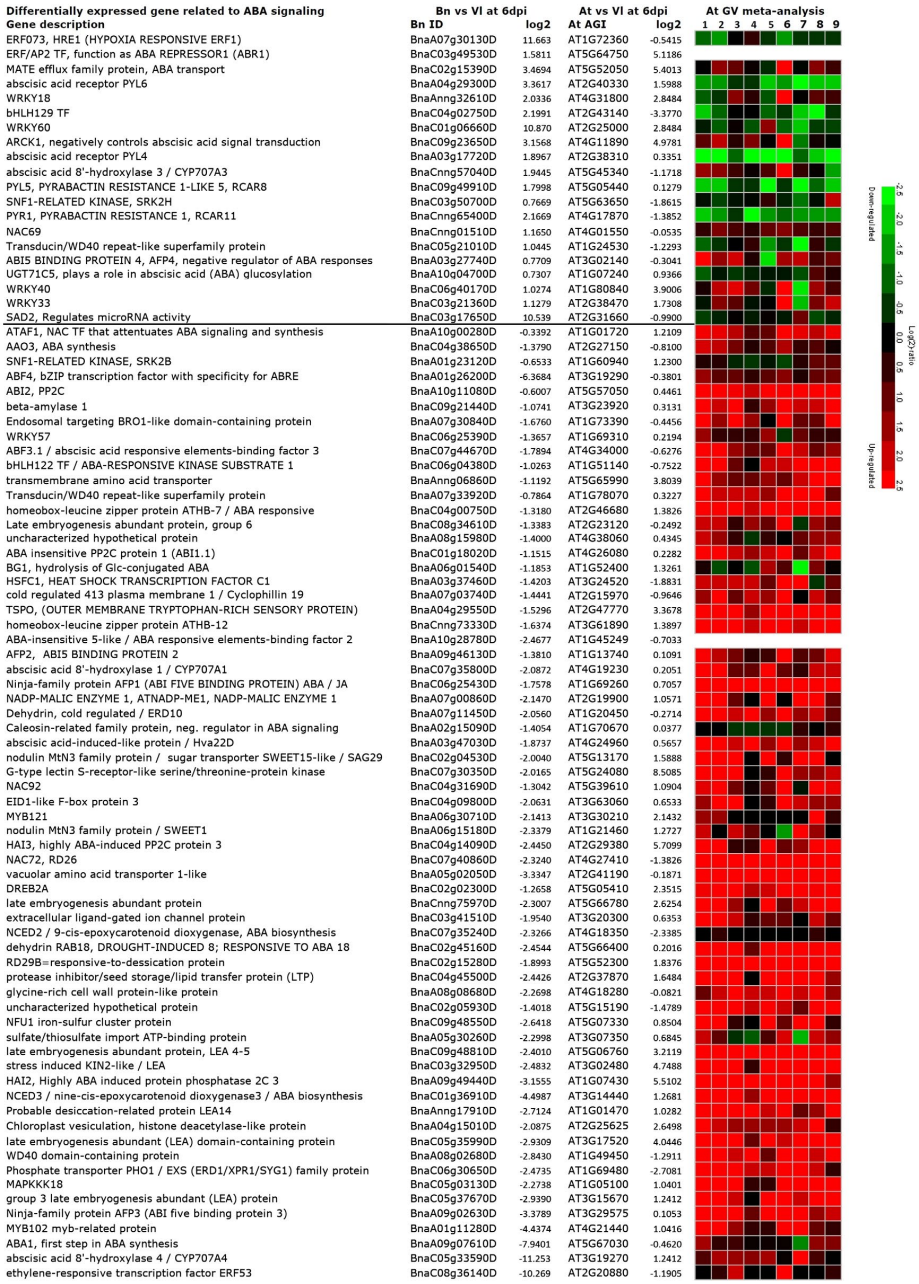
Differentially expressed genes related to abscisic acid (ABA) signalling as identified by RNA‐Seq of *Verticillium longisporum (Vl43)*‐infected oilseed rape (Bn) and *Arabidopsis* (At) roots at 6 days post‐inoculation. The line separates up‐and down‐regulated genes in response to the *Vl43* infection of *Brassica napus*. The meta‐analysis of the orthologous *Arabidopsis* counterparts in response to various abiotic stress treatments focuses on ABA (1: AT‐00110, 2: AT‐00420), high light (3: AT‐00246), cold (4: AT‐00640), drought (5: AT‐00560, 6: AT‐00419), heat (7: AT‐00387), osmotic (8: AT‐00120) and salt stress (9: AT‐00534) treatments.

To investigate the early response of oilseed rape plants to the fungal infection, transcriptomic changes in oilseed rape roots in response to infection with *Vl43* were investigated by RNA‐Seq at 6 dpi, in which non‐infected roots served as a control. The scatterplot in Fig. S1 contains all detected unigenes, being up‐regulated with a log_2_‐ratio ≥ 1 or down‐regulated (log_2_‐ratio ≤ −1). In total about 20% of all identified unigenes were differentially expressed at 6 dpi in oilseed rape as compared to the control (Fig. S1 and Table S1). Furthermore, we also analysed the *Arabidopsis* transcriptome after 6 dpi with *Vl43* from root material by RNA‐Seq (Fig. S1 and Table S1). Differentially expressed genes (DEGs) were classified upon gene ontology (GO) analysis using KOBAS (Table S2; Wu *et al.*, 2006) and the 25 most significantly affected biological processes are displayed in Table 1 (see Table S3 for the complete list). Intriguingly, GO terms for responses to ABA and ABA biosynthesis were one of the most affected biological processes in *B. napus* (Table 1); thus, we investigated the general biosynthesis of the phytohormones SA, JA, ET and ABA upon the GO terms in more detail (Fig. 1), revealing a significant difference between the plant species with respect to ABA biosynthesis. Following this, we searched for more ABA‐related genes in the whole oilseed rape RNA‐Seq dataset with the help of co‐expression analysis by an extensive ATTED‐II analysis with the database of closely related model plant *Arabidopsis* (Fig. S2; Obayashi *et al*., 2007). This led to the identification of 85 potentially ABA‐related DEGs (Fig. 2/Fig. S9). A meta‐analysis (Hruz *et al.*, 2008) of the *Arabidopsis* homologues of these ABA‐related genes confirmed the involvement of the selected DEGs in diverse ABA‐mediated stress responses such as cold, drought, heat and salt treatments. Most (65) of the 85 ABA‐related genes that were suppressed at the early infection by *Vl43* were found to be highly up‐regulated by ABA and abiotic stress treatments (Fig. 2/Fig. S9). The opposite can be observed only for several of the 20 *Vl43* up‐regulated genes, which are displayed above the line in Fig. 2 (Fig. S9). Some of these genes have been described to function as negative regulators in ABA signalling, which could contribute to the overall down‐regulation of ABA responses.

**Table 1:** GO enrichment analysis showing the 25 most significantly enriched GO terms for biological processes derived from all differentially expressed genes (DEGs) (log_2_ ≥ |1|) for *Brassica napus* and *Arabidopsis thaliana*

*Brassica napus*		*Arabidopsis thaliana*	
GO term	*P*‐value	GO term	*P*‐value
Response to abscisic acid	0.00000019	Cell wall organization	0.00000039
Response to hydrogen peroxide	0.00002600	Protein phosphorylation	0.00002800
Response to cadmium ion	0.00009300	Protein autophosphorylation	0.00020000
Response to water deprivation	0.00012000	TM tyrosine kinase signalling pathway	0.00076000
Response to high light intensity	0.00033000	Secondary metabolite biosynthesis	0.00208000
Response to salt stress	0.00049000	Peptidyl‐serine phosphorylation	0.00289000
Seed oilbody biogenesis	0.00049000	Signal transduction	0.00349000
Reductive pentose‐phosphate cycle	0.00091000	Chlorophyll biosynthetic process	0.00436000
Protein sumoylation	0.00141000	Regulation of monopolar cell growth	0.00719000
Photosynthesis, light harvesting in PS I	0.00160000	Root hair cell differentiation	0.00840000
Cell differentiation	0.00183000	Ion transport	0.00965000
Protein import into chloroplast stroma	0.00240000	Pectin catabolic process	0.00987000
Protein stabilization	0.00261000	Cell wall organization or biogenesis	0.00992000
Non‐photochemical quenching	0.00274000	Proteolysis/protein catabolic process	0.01020000
Vegetative/reproductive phase transition	0.00315000	Regulation of pollen tube growth	0.01190000
Abscisic acid biosynthetic process	0.00360000	Response to light stimulus	0.01407000
Lipid storage	0.00373000	Cellular response to iron ion starvation	0.01564000
Lignin biosynthetic process	0.00376000	DNA replication initiation	0.01604000
Response to heat	0.00427000	Cell wall modification	0.01674000
Protein–chromophore linkage	0.00452000	Base‐excision repair	0.01699000
Carbohydrate biosynthetic process	0.00486000	Intracellular signal transduction	0.01797000
Cotyledon vascular tissue formation	0.00529000	Chloroplast fission	0.01859000
Purine ribonucleoside metabolism	0.00643000	Cellular calcium ion homeostasis	0.01931000
Response to desiccation	0.00684000	Terpenoid biosynthetic process	0.01967000
Response to cytokinin	0.00784000	Lipid storage	0.02079000

To verify the RNA‐Seq data, we selected some significantly expressed marker genes for several phytohormone pathways from the RNA‐Seq data and comparatively analysed their expression profiles in three independent biological replicates by RT‐qPCR. To enable an easier data comparison, we calculated the log_2_ values for the qPCR results and confronted them with the RNA‐Seq data. As shown in Table S4 the RNA‐Seq data could be generally confirmed.

### ABA biosynthesis is attenuated in infected oilseed rape roots

**Figure 3:**
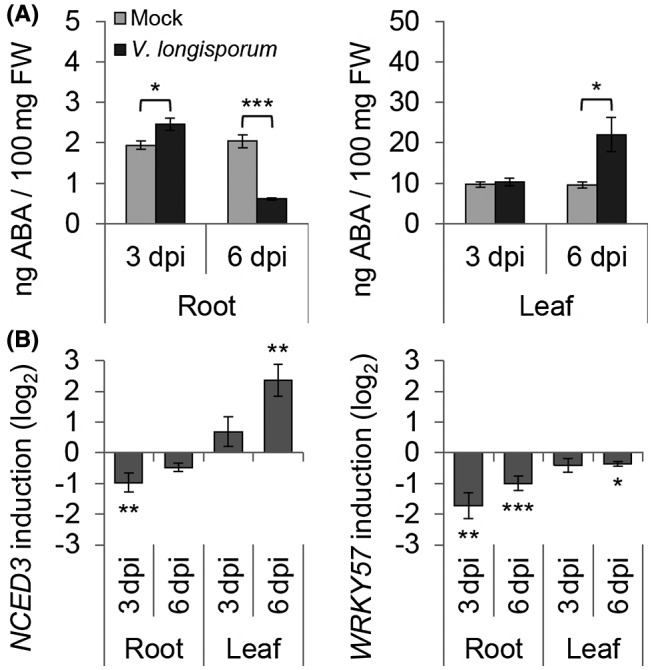
Abscisic acid (ABA) regulation on *Verticillium longisporum (Vl43)* infection in *Brassica napus*. Plant material of *in vitro*‐grown mock‐ and *Vl43*‐treated oilseed rape roots and leaves was sampled at 3 and 6 days post‐inoculation (dpi). (A) Competitive ELISA was applied to quantify the amount of ABA in 100 mg FW plant tissue. (B) RT‐qPCR analysis of the ABA biosynthesis key gene *NCED3* and one of its transcriptional activators, *WRKY57*. Relative expression (log_2_) to *PP2A/Actin2* was calculated with efficiency‐corrected Cq values and the induction (*Vl43* vs mock) in response to the inoculation is displayed for each gene. Error bars indicate the standard error of the mean for three independent biological repetitions and statistics were performed using Student’s *t*‐test (*P ≤ 0.05; **P ≤ 0.01; ***P ≤ 0.001), comparing mock‐ and *Vl43*‐treated samples, respectively.

The overall suppression of the ABA biosynthesis process at 6 dpi in oilseed rape roots implies an attenuated ABA production during the early interaction with *V. longisporum*. To facilitate the characterization of the early plant–fungus interactions, we established an ‘*in vitro*’ infection system, which allows a more accurate monitoring of the infection process and facilitated the generation of equally infected roots (Fig. S3). In Fig. S3A we show that *Vl43* infection does not cause such severe symptoms in oilseed rape as observed in *Arabidopsis* (Fig. S4A), even though in both cases the fungus had successfully entered the plant after 3–4 dpi (Figs S3B and S4B). In a next step, we determined the ABA levels in leaf and root tissues at 3 and 6 dpi, while non‐infected plants served as controls. As shown in Fig. 3A, the basal ABA level remained relatively constant in the non‐infected roots and leaves (~2 ng ABA/100 mg fresh weight (FW) and ~10 ng ABA/100 mg FW, respectively) at 3 and 6 dpi. In response to the fungal infection ABA levels in the leaves were elevated approximately two‐fold. However, in root tissue we initially observed a slight increase at 3 dpi, but at 6 dpi the ABA level in oilseed rape was found to be strongly reduced (Fig. 3A), which is consistent with the RNA‐Seq data from 6 dpi. In addition, we analysed the expression profiles of the ABA biosynthesis gene *NCED3* and one of its transcriptional activators, *WRKY57*, in response to the fungal infection (Fig. 3B). The expression patterns of the both genes behaved largely analogous to the dynamic changes in the ABA content in oilseed rape leaves. Notably, *NCED3* and *WRKY57* were already suppressed in roots at 3 dpi. These data suggest that in oilseed rape roots the ABA content was reduced at the early infection stage mainly via the suppression of ABA biosynthesis genes.

### ABA biosythesis is not affected by *Vl43* infection in *Arabidopsis*

**Figure 4:**
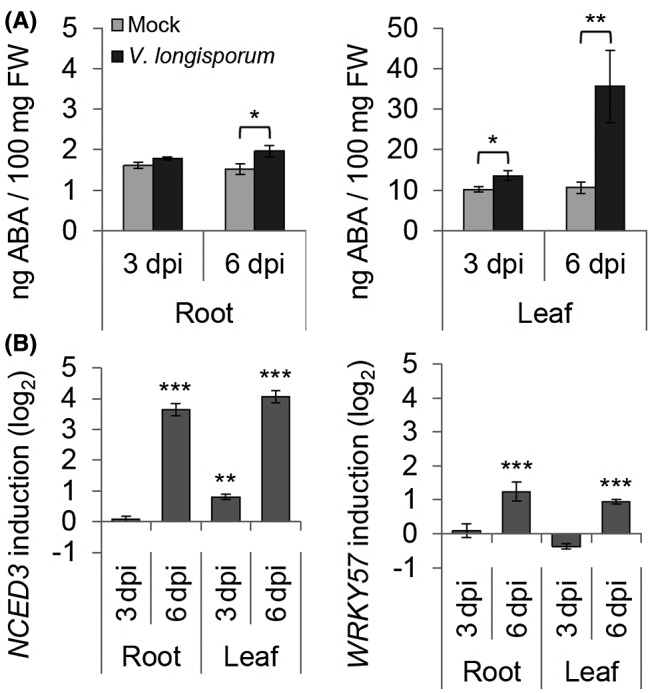
Abscisic acid (ABA) regulation on *Verticillium longisporum (Vl43)* infection in *Arabidopsis thaliana*. Plant material of *in vitro*‐grown mock‐ and *Vl43*‐treated oilseed rape roots and leaves was sampled at 3 and 6 days post‐inoculation (dpi). (A) Competitive ELISA was applied to quantify the amount of ABA in 100 mg FW plant tissue. (B) RT‐qPCR analysis of the ABA biosynthesis key gene *NCED3*, one of its transcriptional activators, *WRKY57*. Relative expression (log_2_) to *PP2A/Actin2* was calculated with efficiency‐corrected Cq values and the induction (*Vl43* vs mock) in response to the inoculation is displayed for each gene. Error bars indicate the standard error of the mean for three independent biological repetitions and statistics were performed using Student’s *t*‐test (*P ≤ 0.05; **P ≤ 0.01; ***P ≤ 0.001), comparing mock‐ and *Vl43*‐treated samples, respectively.

In a similar experiment, transcriptomic changes in *Vl43*‐infected *Arabidopsis* were analysed by RNA‐Seq from root material obtained from the ‘*in vitro*’ infection system (Fig. S4). The results are summarized in Table S1. In total, about 30% of unigenes identified were differentially expressed in *Arabidopsis* roots at 6 dpi as compared to the non‐infected control (Fig. S1). By GO annotation these DEGs were analysed for their participation in biological processes (Fig. 1 and Table S2). Differing from oilseed rape, GO terms for responses to ABA and ABA biosynthesis were not significantly enriched in *Arabidopsis*. Furthermore, the comparison of the expression patterns of all DEGs assigned to the GO terms for biosynthesis of the phytohormones SA, JA, ET and ABA differed between oilseed rape and *Arabidopsis* roots (Fig. 1). While SA biosynthesis is predominantly induced in oilseed rape, JA/ET biosynthesis appears to be up‐regulated in *Arabidopsis*. Most notably, genes associated with ABA biosynthesis were not found to be collectively suppressed in *Arabidopsis* roots at 6 dpi (Figs 2 and 4B). By contrast to oilseed rape, ABA levels significantly increased in both roots and leaves of *Arabidopsis* on *Vl43* infection (Fig. 4A), Accordingly, we did not observe down‐regulation of the ABA synthesis gene *NCED3* and the transcriptional activator *WRKY57* (Fig. 4B).

### Suppression of ABA‐related genes goes along with an inhibition of the JA/ET pathway in oilseed rape roots

**Figure 5:**
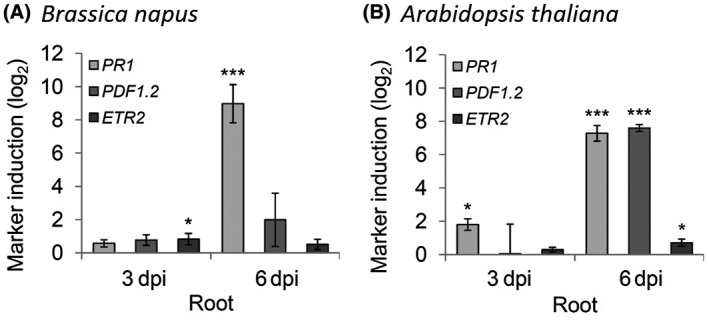
Effects on main defence hormone pathway marker gene expression. The expression of three typical phytohormone marker genes was investigated by RT‐qPCR in root material from *Brassica napus* (A) and *Arabidopsis thaliana* (B). *PR1* for SA, *PDF1.2* for JA and *ETR2* for ET. Relative expression (log_2_) to *PP2A/Actin2* was calculated with efficiency‐corrected Cq values and the induction (*Vl43* vs mock) in response to the inoculation is displayed for each gene. Error bars indicate the standard error of the mean for three independent biological repetitions and statistics were performed using Student’s *t*‐test (*P ≤ 0.05; **P ≤ 0.01; ***P ≤ 0.001), comparing mock‐ and *Vl43*‐treated samples, respectively.

To investigate the impact of the ABA‐response suppression on other phytohormone pathways, we analysed the expression levels of three typical phytohormone marker genes *PR1* (SA marker), *PDF1.2* (JA marker) and *ETR2* (ET marker) in response to the fungal infection (Fig. 5). At 3 dpi we observed only for *ETR2* a slight induction (0.82‐fold log_2_ induction; *P* = 0.011) in oilseed rape roots, while at 6 dpi the expression of *PR1* was strongly induced (8.97‐fold log_2_ induction; *P* < 0.001). However, the expression level of *PDF1.2* was not significantly increased and no significant induction was observed for *ETR2* at 6 dpi (Fig. 5A). These results suggest that *V. longisporum* might be able to interfere with the phytohormone crosstalk, e.g. via suppression of the NCED3‐mediated ABA biosynthesis accompanied with damped JA/ET responses at the early infection stage. We investigated the same phytohormone marker genes in infected *Arabidopsis* roots (Fig. 5B) and found *PR1* already significantly induced at 3 dpi and further increased to a 7.3‐fold log_2_ induction at 6 dpi (*P* < 0.001). Additionally, *PDF1.2* was strongly induced (7.59‐fold log_2_ induction; *P* < 0.001) at 6 dpi and the ethylene marker *ETR2* was slightly (0.71‐fold log_2_ induction; *P* = 0.049) up‐regulated at the 6 dpi time point, which clearly differed from oilseed rape.

### ABA‐deficient *Arabidopsis* mutants display reduced symptom development

**Figure 6:**
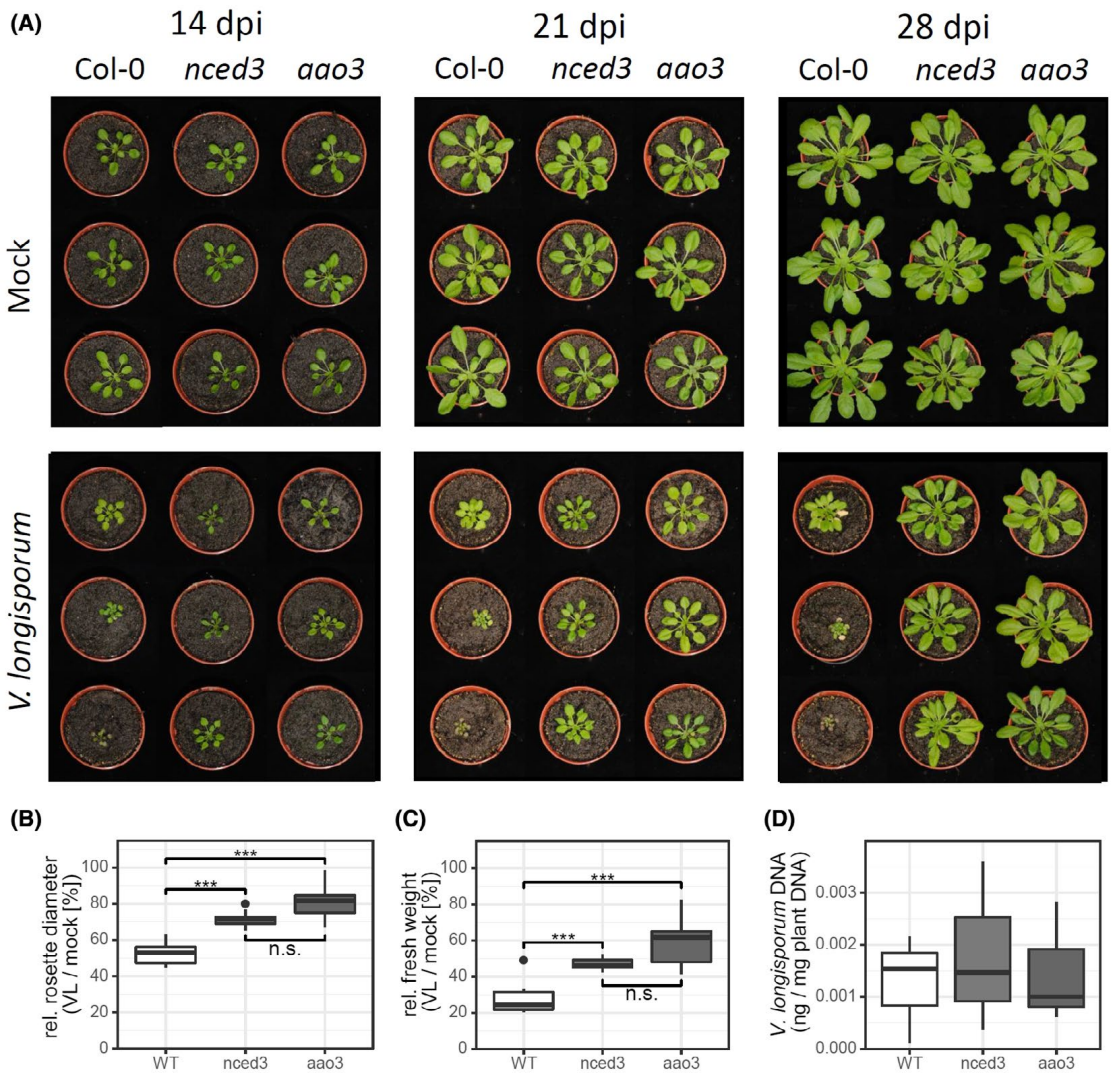
The *Arabidopsis* *nced3* and *aao3* mutants show delayed development of disease symptoms. Plants grown on sand/soil were photographed at the indicated time points (A). In an independent experiment plants were sampled at 24 days post‐inoculation (dpi) and the rosette diameter (B), the fresh weight (C) and relative amount of fungal DNA (D) were determined as described in the Experimental Procedures. Multiple comparison of means was performed by ANOVA and a post hoc Tukey test (*P ≤ 0.05; **P ≤ 0.01; ***P ≤ 0.001).

**Figure 7:**
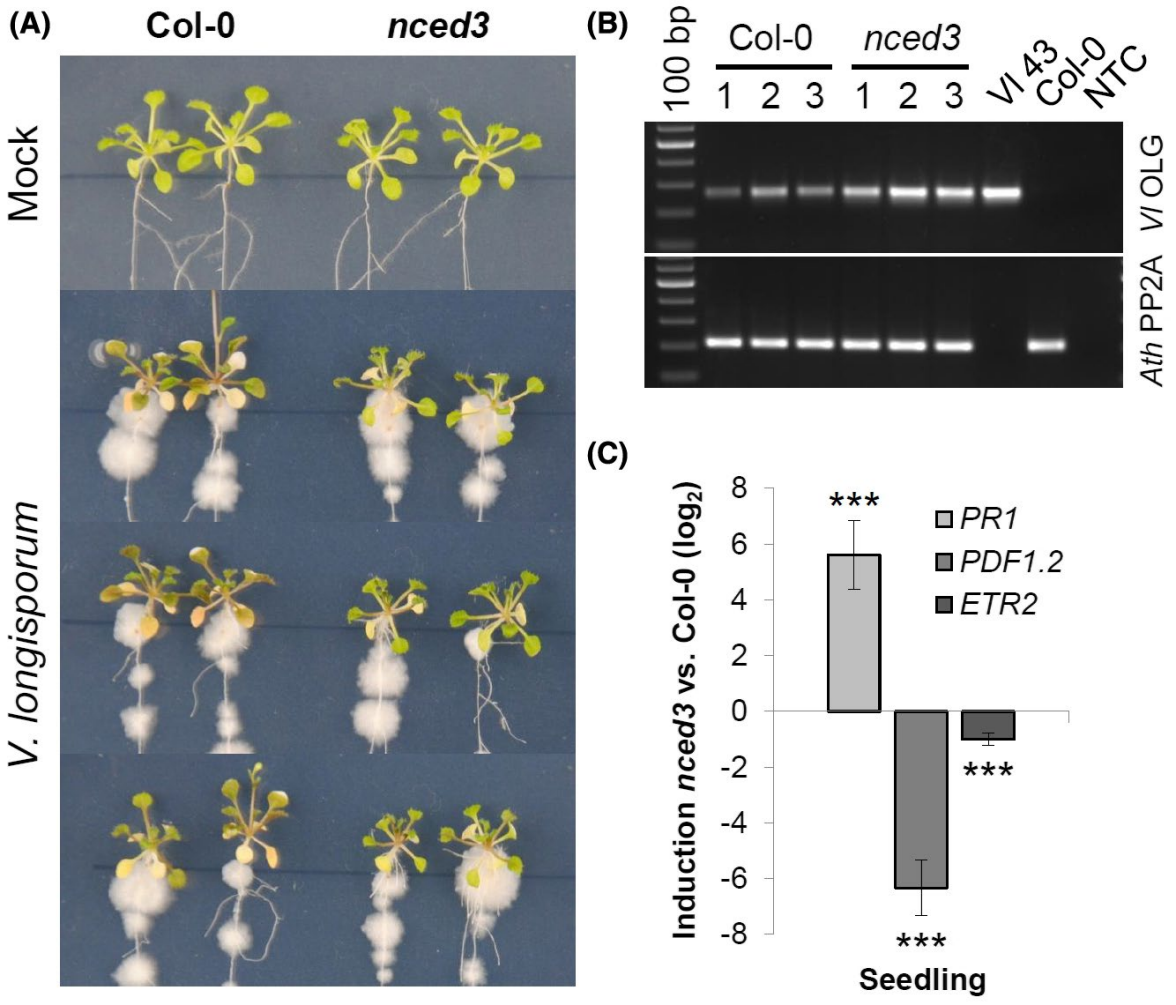
*In vitro* grown *Arabidopsis* *nced3* plants show delayed disease symptom development and constitutively altered hormone marker expression. (A) *Arabidopsis* seedlings were grown on MS plates and photographed at 12 days post‐inoculation (dpi), indicating that *nced3* knockout plants are more tolerant to *Verticillium longisporum (Vl43)* infection compared to Col‐0 wild‐type plants. (B) The infection progress was monitored by the amount of fungal DNA determined by semiquantitative PCR using leaf material harvested at 12 dpi. Comparing fungal amplicon signals to plant *PP2A* signals indicates that fungal growth was rather enhanced in the *nced3* mutant. (C) Expression analysis of *PR1* for salicylic acid, *PDF1.2* for jasmonic acid and *ETR2* for ethylene in the *Arabidopsis* *nced3* mutant. Material of *in vitro*‐grown 3‐week‐old *Arabidopsis* seedlings of *nced3* and Col‐0 was sampled. Relative expression (log_2_) to *PP2A/Actin2* was calculated with efficiency‐corrected Cq values and the induction (*nced3* vs Col‐0) in response to the *nced3* knockout is displayed for each gene. Error bars indicate the standard error of the mean for three independent biological repetitions and statistics were performed using Student’s *t*‐test (*P ≤ 0.05; **P ≤ 0.01; ***P ≤ 0.001), comparing *nced3* and Col‐0 samples, respectively.

To clarify the role of ABA in the plant–fungus interaction, we challenged the ABA biosynthesis mutants *nced3* and *aao3* with the *Vl43* infection, and compared the results with Col‐0 wild‐type plants with respect to disease progression and symptom development. As compared to Col‐0, both *nced3* and *aao3* knockout mutant plants grown on soil displayed reduced symptoms as well. At 21 dpi, the stunted shoot growth and senescence‐like symptoms became obvious in the wild‐type plants, but were less pronounced in the *nced3* and *aao3* mutant. At 28 dpi, the wild‐type plants severely suffered from the infection and displayed strong growth depression as well as senescence‐like symptoms, while the *nced3* and *aao3* were much less affected, suggesting a delayed/impaired disease progression (Figs 6A and 7A). Comparing infected with uninfected plants, the relative rosette diameter and fresh weight were significantly enhanced in both mutants compared to Col‐0 (Fig. 6B,C). Although symptoms in the mutants were much less pronounced, the fungal biomass was not reduced as compared to the wild‐type (Figs 6D and 7B). These data demonstrate that in *Arabidopsis* the impaired ABA biosynthesis strongly affects symptom development, but does not negatively affect fungal colonization. In addition, we observed that external application of ABA on oilseed rape appears to impair the fungal infection progression, resulting in a reduced hypocotyl colonization as measured by determination of fungal DNA within hypocotyls (Fig. S8B,C), albeit data were not statistically significant due to a high variation in fungal infection and low case number in this preliminary experiment. Although ABA application at 6 dpi did not yet show altered symptoms, the observed tendency in decreased fungal colonization is consistent with observations that the *Arabidopsis* ABA mutants showed enhanced tolerance to *Vl43* (Figs 6D and 7B).

To understand possible mechanisms underlying the *nced3*‐mediated tolerance, we compared the expression levels of the SA, JA and ET marker genes in the *nced3* mutant and Col‐0 wild‐type plants. As shown in Fig. 7C, the *nced3* mutant displayed constitutively higher *PR1* levels, while *PDF1.2* and *ETR2* were both strongly suppressed as compared to Col‐0, suggesting that the suppression of ABA biosynthesis affects other phytohormone pathways in the *nced3* mutant in a way similar to that observed in *Vl43*‐infected oilseed rape roots (Figs 5 and 7C). The lack of a functional *NCED3* gene in the *Arabidopsis* mutant obviously promoted SA signalling while reducing JA/ET signalling. This could be a reason for the tolerance to fungal infection together with delayed disease symptoms and is in agreement with our observation that *PDF1.2* expression was attenuated after 6 dpi in *B. napus* as compared to *Arabidopsis* (Fig. 5).

## Discussion

To shed light on the early molecular events in the plant–*V. longisporum* interaction, we investigated transcriptomic changes at 6 dpi in *B. napus* and *A. thaliana* roots by RNA‐Seq experiments. The generated RNA‐Seq data provided us with a comprehensive view of molecular plant–*Verticillium* interactions. Surprisingly, most significantly enriched GO terms differed between oilseed rape and *Arabidopsis*, especially with respect to ABA signalling and biosynthesis (Table 1). ABA‐biosynthesis related genes were overall down‐regulated and ABA levels were accordingly decreased in oilseed rape roots at 6 dpi (Figs 1 and 2). Although interactions of *Vl43* with both oilseed rape and *Arabidopsis* are compatible, we observed that infected *B. napus* plants display a less diseased phenotype (Figs S3A, S4A and S5), leading to the assumption that differential ABA signalling plays a pivotal role in the mediation of symptomless infection in oilseed rape within the early infection stage. While the ABA level more or less increased in leaves of oilseed rape, it was found to be significantly reduced in the roots at 6 dpi (Fig. 3A). These data underpin the RNA‐Seq results, reinforcing the hypothesis of a pivotal role for ABA in regulating the early plant–fungus interaction in oilseed rape. Interestingly, in accordance with the RNA‐Seq data, a reduction in ABA levels was not observed in the infected *Arabidopsis* roots, where the ABA levels by contrast appear to be gradually elevated during the infection progression (Fig. 4A). This observation is congruent with reports from Iven *et al. *(2012), who reported an increase in ABA levels in *V. longisporum*‐infected *Arabidopsis* roots at 6 and 8 dpi. It was furthermore shown that in *Arabidopsis* leaves ABA increased at 2 dpi (Roos *et al.*, 2014) and also later at 15 dpi in *Arabidopsis* petioles in response to *V. longisporum* (Ralhan *et al.*, 2012). The increase in ABA in the leaves of oilseed rape and *Arabidopsis* likely results from side effects, provoked by the fungal invasion into the roots and growth in the xylem, which could lead to an initially reduced water supply within the leaves. However, Reusche *et al. *(2012) reported that at 7 dpi *V. longisporum* triggers *de novo* xylem formation that can compensate for compromised water transport in brassicaceous host plants, which can explain why this vascular pathogen does not affect drought stress responses in oilseed rape (Lopisso *et al.*, 2017). Furthermore, the increased ABA levels in *Arabidopsis* might contribute to an increased drought stress tolerance that has been observed for *V. longisporum*‐infected plants (Reusche *et al.*, 2014).

In plants, the ABA level can be influenced by blocking ABA biosynthesis, by ABA inactivation or by degradation. In addition to suppressed ABA biosynthesis‐related genes in the RNA‐Seq data, some ABA catabolic genes described by Finkelstein (2013) have also been identified, e.g. *CYP707A3* is up‐regulated, while *CYP707A1* is down‐regulated (Fig. 2). Also, two genes involved in ABA inactivation (*UGT71C5* and *BG1*; Liu *et al*., 2015b) were found in the RNA‐Seq data, which were up‐ and down‐regulated by the fungal infection, respectively (Fig. 2). Several ABA‐related genes up‐regulated in the *B. napus* response to *V. longisporum* infection have been described as negative regulators of the ABA response, e.g. WRKY18, WRKY33, ARCK1 and bHLH129. Others encode ABA receptor components (e.g. PYL4, 5 and 6), which might render the plant more sensitive to low ABA levels. Several ABA‐responsive genes containing the ABRE (C/TACGTGG/TC) and DRE (A/GCCGAC) *cis*‐elements in their promoters, including *RD29B*, *NAC072*, *Hva22c*, *ERD10*, *LEA14* and *RAB18* (Nakashima *et al.*, 2014; Yoshida *et al.*, 2014), were also found to be suppressed in oilseed rape (Fig. 2). Following this, we conclude that the infection resulted in a drop in ABA levels in the infected oilseed rape roots primarily by reducing ABA biosynthesis via suppression of gene expression of this process.

The RNA‐Seq data and qPCR experiments show that the expression levels of *NCED3*, a key gene involved in stress‐induced ABA biosynthesis (Jensen *et al.*, 2013), and *WRKY57*, one positive regulator of *NCED3* (Jiang *et al.*, 2012), were both suppressed in the 6 dpi oilseed rape roots, supporting a crucial role of *NCED3* for ABA biosynthesis (Fig. 3B). As the expression profile of *NCED3* correlated mostly with that of *WRKY57*, we believe that suppression of ABA production is initiated upstream of this transcription factor in oilseed rape. Accordingly, this suppression is absent in the infected *Arabidopsis* roots in which both genes were up‐regulated (Fig. 4B), suggesting the involvement of the *NCED3‐*mediated ABA biosynthesis specifically in the early interaction of *Vl43* and oilseed rape. Genetic evidence for a crucial role of *NCED3‐*mediated ABA biosynthesis in the plant–*V. longisporum* interaction came from the analysis of two *Arabidopsis* ABA‐deficient mutants, *nced3* and *aao3*, both appearing to tolerate *Vl43* colonization as compared to the wild‐type control, suggesting that ABA is required for full symptom development of *Arabidopsis* in response to *Vl43* infection.

Since ABA is often reported to play a role in attenuating defence reactions towards biotic stress (Cao *et al.*, 2011; Clay *et al.*, 2009; Mialoundama *et al.*, 2009), attempts to elevate ABA levels might be a genuine strategy for pathogens to manipulate their hosts. This can occur by producing ABA themselves (Cao *et al.*, 2011; Morrison *et al.*, 2015; Siewers *et al.*, 2006; Spence *et al.*, 2015; de Torres‐Zabala *et al*., 2015) or by activation of the plant ABA biosynthesis, for example via enhancing *NCED3* gene expression as observed in the *X. oryzae*–rice interaction, resulting in increased susceptibility (Xu *et al.*, 2013). Similar observations have been made for *Pseudomonas syringae* DC3000, which deploys type III effectors to induce the *Arabidopsis* *NCED3* gene, resulting in elevation of ABA levels (de Torres‐Zabala *et al.*, 2007, 2009). However, some *Arabidopsis* ABA mutants were more susceptible towards *Pythium irregulare* and *A. brassicicola,* thus ABA is believed to benefit plant responses against necrotrophic pathogens via enhancement of the JA response (Lorenzo *et al.*, 2004). Accordingly, we found that the JA/ET response was compromised in oilseed rape when comparing the *PDF1.2* and *ETR2* expression level with that in *Arabidopsis*. Furthermore, we observed an elevated *PR1* transcript accumulation that in *Arabidopsis* already started at 3 dpi was strongly induced at 6 dpi in both oilseed rape and *Arabidopsis* (Fig. 7). While SA, JA and ET can be negatively affected by ABA (Cao *et al.*, 2011; Fan *et al.*, 2009; Mauch‐Mani and Mauch, 2005; Ton *et al.*, 2009), plants can attenuate ABA signalling through the action of SA/biotic stress‐inducible WRKY33 (Liu et al., 2015a) or via ET signalling (Anderson *et al.*, 2004; Arc *et al.*, 2013). Thus, the reciprocal antagonism between ABA and SA (Yasuda *et al.*, 2008) might be the reason for ABA suppression in oilseed rape infected with *V. longisporum*. However, since the SA pathway appears to be activated in both plant species upon infection with *V. longisporum*, this phytohormone is likely not responsible for suppression of ABA in oilseed rape, although SA has been reported to exert a negative impact on ABA (Yasuda *et al.*, 2008). On the contrary, the reduced ABA levels in *B. napus* could facilitate the relatively stronger induced expression of *PR1* and SA biosynthetic genes in oilseed rape as compared to *Arabidopsis* at 6 dpi (Figs 1 and 5). It has been demonstrated that ABA can suppress PAL‐dependent SA biosynthesis (Cao *et al.*, 2011) and promotes susceptibility in the rice–*X.oryzae* interaction by suppressing SA‐mediated defences (Xu *et al.*, 2013). The SA increase in turn might then result in an attenuated expression of *PDF1.2* (Thaler *et al.*, 2012) and *ETR2*, which is consistent with our finding that in the *nced3* mutant both *PDF1.2* and *ETR2* were suppressed while the *PR1* was induced (Fig. 7C). It is therefore reasonable to speculate that this kind of phytohormone crosstalk at the early infection stage is fully employed in the decelerated and delayed disease symptom development in oilseed rape as compared to *Arabidopsis*. A similar pattern of differential marker expression for *PR1* and *PDF1.2* in *B. napus* roots was also reported by Kamble *et al.* (2013).

Interestingly, it appears that the fungal colonization is not impaired in *A. thaliana* ABA biosynthesis mutants as evidenced by similar amounts of fungal biomass in *nced3* and *aao3* plants as compared to *Arabidopsis* wild‐type plants. However, the external ABA application to oilseed rape reduced fungal colonization in hypocotyls as measured at 6 dpi, suggesting an impaired infection progression. This is in agreement with our hypothesis that the fungus may benefit from reduced ABA levels (Fig. S8). Whether ABA suppression is part of the plant defence response differing between oilseed rape and *Arabidopsis* or due to a host‐specific fungal influence is currently not clear and a highly intriguing question. As both plant species, *B. napus* and *Arabidopsis*, are susceptible to *Vl43* infection, the observed up‐regulation of genes or accumulation of hormones might be caused by the pathogen infection process rather than by the plant defence response, which generally does not appear to be effective enough to restrict the disease progress. It has been demonstrated that in *Arabidopsis* the more resistant Bur ecotype showed a stronger induction of SA after *V. longisporum* infection than the more susceptible Ler ecotype, in which the level of ABA was higher (Häffner *et al.*, 2014). Veronese *et al *(2003) showed that the *Arabidopsis* *aba2‐1* mutant displayed reduced disease symptoms (anthocyanin accumulation) towards *V. longisporum* infection as compared to wild‐type plants (the strain Bob.70, 90‐02 was originally assigned to *Verticillium dahliae*; Inderbitzin *et al.*, 2011, Table S2). These results are congruent with our findings that lower ABA levels go along with increased SA responses and result in reduced symptom development. This is further supported by analysis of the ABA mutants *ahg2‐1* and *aba1‐6*, in which the SA pathway was constitutively activated, also conferring some resistance to the necrotrophic pathogens *B. cinerea* (Nishimura *et al.*, 2009) and *Plectosphaerella cucumerina* (Sanchez Vallet *et al*., 2012), respectively.

**Figure 8:**
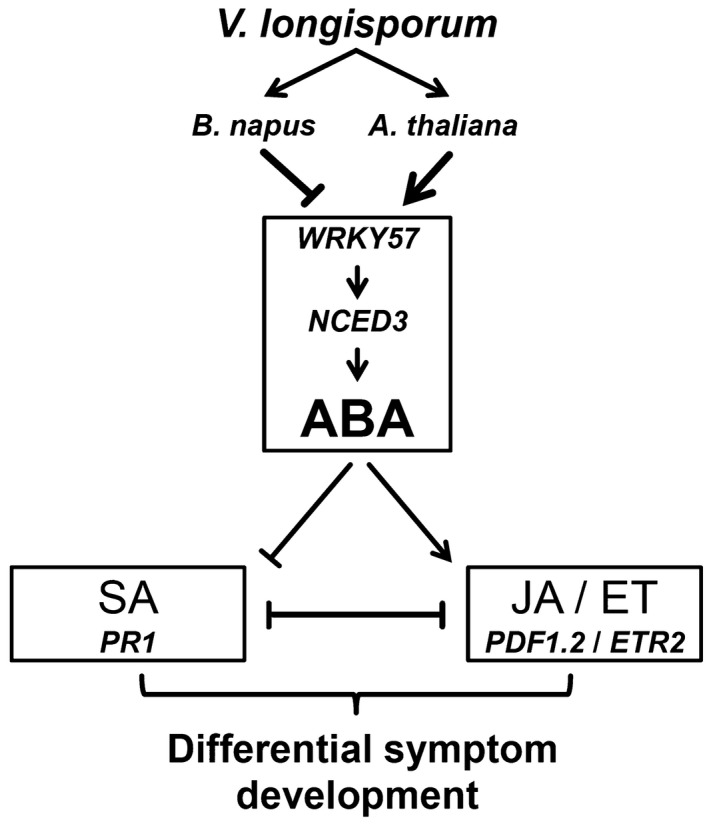
Simplified view of abscisic acid (ABA) signalling responses to the infection with *Verticillium longisporum (Vl43)* in oilseed rape and *Arabidopsis* roots at the early infection stages. ABA is functionally involved in plant–*Vl43* interaction, which is different between oilseed rape and *Arabidopsis* at the early infection stages. *Vl43* attenuates ABA signalling in oilseed rape roots by interference with *NCED3*‐mediated ABA biosynthesis triggering an enhanced salicylic acid (SA) and concomitantly impaired jasmonic acid (JA) and ethylene (ET) responses, which results in delayed symptom development. In *Arabidopsis*, JA/ET responses are not affected and full symptom development can be observed. The possible fungus‐induced suppression of ABA in oilseed rape may represent a specific virulence mechanism, allowing the fungus to successfully spread in the plant and coexist with its host in a longer lasting compatible interaction.

Our data indicate that in oilseed rape SA biosynthesis is predominantly induced, while in *Arabidopsis* JA/ET biosynthesis is mainly up‐regulated on *Vl43* infection. This discrepancy might be a hint for a specific evolutionary co‐existence of oilseed rape and *V. longisporum* as discussed by Eynck *et al. *(2009) and Inderbitzin *et al. *(2011). The fungus probably manipulates ABA signalling in oilseed rape roots in order to prevent a strong JA/ET resistance response and thereby establish a longer lasting compatible interaction with its host (coexistence). This would be in agreement with findings by Roos *et al. *(2015), who found for *Arabidopsis* that JA and MYC2 are required for full induction of TPS23/TPS27‐dependent production of monoterpenes, which stimulate germination and enhance *V. longisporum* invasion. Consequently, *myc2‐1* mutants display higher resistance to *V. longisporum*. According to Anderson *et al. *(2004), MYC2 is responsive to both JA and ABA. In addition to down‐regulation of the positive regulator *WRKY57*, we observed differential expression of additional negative regulators of ABA signalling, including *WRKY18* (Rushton *et al.*, 2012; Shang *et al.*, 2010; Xu *et al.*, 2006), *WRKY33* (Liu *et al*., 2015a) and *bHLH129* (Tian *et al.*, 2015), which were all up‐regulated at 6 dpi in oilseed rape roots. Furthermore, two NAC transcription factors, which were previously reported to be involved in ABA‐related responses, *NAC72* and *NAC69* (Fujita *et al.*, 2004; Park *et al.*, 2011), were down‐ and up‐regulated, respectively. Thus, we expect a much more complex network of transcription factors being involved in this regulation than presented in our summarizing model, which focuses for clarity on WRKY57‐ and NCED3‐dependent ABA biosynthesis (Fig. 8).

In conclusion, this study provides a molecular insight into early oilseed rape–*V. longisporum* interactions pointing to an important involvement of reduced ABA levels. In addition, our results constitute a unique platform for further characterizing and understanding this infection process, which may be useful for breeding of resistant oilseed rape varieties against *V. longisporum* infection.

## Experimental Procedures

### Fungal cultivation

*Verticillium longisporum* isolate *Vl43* was provided by Dr Elke Diederichsen (FU Berlin, Germany) in the form of malt extract agar plates inoculated with the fungus. Glycerol stock cultures (22%) of *Vl43* conidia were produced by transferring mycelium plugs into liquid Czapek–Dox medium. After 1 week growing in the dark on a rotating shaker, conidia were harvested with the help of a gauze filter (mesh 200 µm; Hydro‐Bios Kiel, Germany). *Verticillium* conidia from glycerol stocks were spread on potato dextrose agar (PDA) plates and grown for 2 weeks at room temperature (RT) in the dark. Plugs of these plates were again transferred to liquid Czapek–Dox medium and incubated on a rotary shaker for 3 days at RT in the dark to massively produce conidia. After 3 days, conidia were filtered through gauze, centrifuged for 8 min at 8000 rpm and resuspended in sterile Czapek–Dox medium to increase the concentration. Stocks for infection were supplemented with 86% glycerol to a final concentration of 22% and kept at −80 °C till the day of inoculation. At the day of inoculation stocks were thawed, centrifuged for 8 min at 8000 rpm and resuspended in tap water (‘*in vivo*’ infection) or sterile Czapek–Dox medium (‘*in vitro*’ infection) to a final concentration of 2 × 10^6^ conidia/mL. Tap water and Czapek–Dox medium were used as mock treatments, respectively.

### Plant material

Seeds of *B. napus* 'Express' (susceptible reference line #617 from NPZ Hans‐Georg Lembke KG, Holtsee, Germany), *A. thaliana* Col‐0 and the mutant lines (see below) were surface sterilized with 6% sodium hypochlorite/Tween 20 solution and 70% ethanol followed by washing (three times) with sterile distilled water.

### Infection on soil of *B. napus* and *A. thaliana*

Sterilized seeds were first grown *in vitro* on ½ MS medium (0.5% sucrose) under short day conditions (8 h light, 22 °C). *A. thaliana* plants were grown in Petri dishes for 14 days whereas *B. napus* plants were grown in 250 mL salad cups for 7 days. At the day of inoculation plants were removed from the medium and root dipped in *Vl43* conidia solution (or mock) for 15 min. *B. napus* plants were then transferred to plastic folding boxes (2 × 1.5 cm) filled with non‐sterile sand (Bauhaus hardware store, Belp, Switzerland) and fertilized with WUXAL® universal fertilizer‐treated tap water (Shen *et al.*, 2014). *A. thaliana* plants were transferred to pots containing non‐sterile sand/soil (1:1) and also supplemented with fertilizer‐treated tap water. All the plants were placed in a climate cabinet (Vötsch VB 0714‐A, Reiskirchen, Germany) with short day conditions. For *B. napus,* roots of 15 infected and 15 non‐infected plants were sampled after 6 days and immediately flash‐frozen in liquid nitrogen. Photographs of *A. thaliana* plants were taken once per week to observe the infection process. At 24 dpi eight infected plants per line were deployed to measure and calculate fresh weight (FW) and diameter of rosettes in relation to the mean of mock‐treated controls. Statistics were calculated by a post hoc Tukey test (*P ≤ 0.05; **P ≤ 0.01; ***P ≤ 0.001).

### Infection on MS plates of *B. napus* and *A. thaliana*

Plants were pre‐grown as described for the *in vivo* infection. After 14 days *A. thaliana* plants were first transferred to 120 × 120 mm Petri dishes containing ½ MS (0.5% sucrose) and grown vertically for another 7 days before being inoculated. Seven‐day‐old *B. napus* plants were removed from the salad cups and placed on 120 × 120 mm Petri dishes containing ½ MS (0.5% sucrose) for direct inoculation and further cultivated under short day conditions. To inoculate the roots a brush was dipped in *Vl43* conidia suspension (2 × 10^6^/mL) or Czapek–Dox medium as mock treatment and used to distribute the conidia along the roots. Roots of *B. napus* plants were inoculated completely, whereas the upper 2 cm of *A. thaliana* roots were spared. Petri dishes were closed and plants were grown vertically in short day conditions. The progress of fungal growth and the infection was observed for the next 12 days (Figs 3A and 4A). Plant material was sampled at 3 and 6 dpi. Roots and leaves of *B. napus* and *A. thaliana* were sampled separately. All samples were immediately frozen in liquid nitrogen.

### *Verticillium* genomic DNA (gDNA) detection in plant tissue

*Brassica napus* hypocotyls and *Arabidopsis* leaves from infected plants were sampled at the indicated time points. Each biological replicate consists of three individual hypocotyls, three *in vitro* *Arabidopsis* shoots or two complete *Arabidopsis* *in vivo* rosettes, respectively. For gDNA isolation the tissue was immediately flash‐frozen in liquid nitrogen and ground using a mortar and pestle. Total gDNA was isolated using cetyltrimethylammoniumbromide (CTAB) buffer (Rogers and Bendich, 1985) supplemented with β‐mercaptoethanol. For semiquantitative PCR amplification Dream Taq Polymerase (Thermo Scientific, Waltham, MA) was used according to the manufacturer's instructions (hypocotyls and *in vitro* shoots). For real‐time qPCR quantification of fungal DNA in rosettes the Maxima SYBR Green/ROX qPCR Master Mix (Thermo Scientific) was used according to the manufacturer's instructions. Primers used for *Vl43* detection (Eynck *et al*., 2007) are listed in Table S7. PCR was performed using the following conditions: 3 min at 95 °C; 35 cycles of 20 s at 95 °C, 20 s at 58 °C and 30 s at 72 °C. For semiquantitative PCR, products were run in a 1.7% agarose gel for 60 min at 75 V. Relative amounts of DNA were calculated by use of Image Lab software (Bio‐Rad, Hercules, CA). The amount of fungal DNA was normalized against *PP2A* signals from plant DNA. Error bars indicate the standard deviation (±SD) of three different biological repetitions and statistics were performed using Student’s *t*‐test (*P ≤ 0.05). For real‐time PCR, standard curves for the quantification of gDNA were calculated by dilution of a series of known concentrations of pure *Arabidopsis* and *Vl43* DNA. For each sample the amount of fungal DNA in relation to plant DNA was calculated in ng/mg.

### *Vl43* hypocotyl assays

To detect *Vl43* within the tissue of *B. napus*, hypocotyls were surface sterilized with a 6% sodium hypochlorite/Tween 20 solution and 70% ethanol for 3 min, followed by washing three times with sterile distilled water. The edges were cut and hypocotyls were placed on PDA plates. A possible fungal colonization of cotyledons and leaves at the observed time points was also checked, but not observed (Fig. S6). Pictures were taken with a stereomicroscope (Zeiss, Stereo Discovery.V20, Oberkochen, Germany) after 4 days.

### *Vl43* detection in *A. thaliana* roots

Roots of *in vitro*‐infected *A. thaliana* Col‐0 plants were sampled at 4 dpi and incubated in 10% KOH at 90 °C for 10 min. After that the samples were washed three times for 15 min in distilled water. Then the samples were stained in 10 µg/mL fluorescein isothiocyanate (FITC)‐wheat germ agglutinin (WGS) (Sigma‐Aldrich, St Louis, MO) and dissolved in 50 mM HEPES buffer, pH 7.5 (Roth GmbH + Co. KG, Karlsruhe, Germany), in the dark for 1 h. After staining the samples were washed three times for 15 min in 50 mM HEPES buffer in the dark. Samples were placed on microscope slides together with 50 mM HEPES buffer containing 2% DABCO and analysed using a confocal laser scanning microscope (Leica TCS SP1, Leica Microsystems, Wetzlar, Germany).

### RNA isolation

Total RNA from different tissues was extracted by TRIzol® (Thermo Fisher Scientific Inc., Waltham, MA) reagent according to the manufacturer’s recommendation. The quality and concentration of RNA was controlled by gel electrophoresis and a NanoVue Plus Spectrophotometer (GE Healthcare Life Science, Chicago, IL).

### RNA‐Seq data processing

Equal amounts of RNA from three biological replicates of mock‐ and *Vl43*‐inoculated soil‐grown oilseed rape roots as well as *in vitro* mock‐ and *Vl43*‐treated *Arabidopsis* roots were pooled for sequencing. Forward/reverse clean reads for *B. napus* and *Arabidopsis* were obtained by Illumina sequencing from Beijing Genomics Institute BGI (Hong‐Kong, China) and Novogene Co., Ltd (Hong‐Kong, China), respectively. Fragments per kilobase of transcript per million mapped reads (FPKM) values for all samples were calculated using the open‐source software tools HISAT and StringTie with default settings, as described by Pertea *et al. *(2016), with the *B. napus* genome (v. 4.1) and annotation (v. 5) (Chalhoub *et al.*, 2014) and the TAIR10 reference genome/annotation (Lamesch *et al.*, 2011) for *Arabidopsis*. GO terms for *B. napus* sequences were annotated by BLASTing the reference peptide library (v. 5) (Chalhoub *et al.*, 2014) against *A. thaliana* reference peptides containing GO and KEGG annotations using KOBAS (Wu *et al.*, 2006). The GO enrichment analysis was performed with R (R core Team, 2018) using the topGO (Alexa and Rahnenfuhrer, 2018) package. The bioinformatics pipeline is summarized in Fig. S7.

### Data deposition

The data reported in this paper have been deposited in the National Center for Biotechnology Information BioProject archive, https://www.ncbi.nlm.nih.gov/bioproject, accession PRJNA419857 for *B. napus* and PRJNA563868 for *A. thaliana*).

### Primer design and gene specificity

A list of the investigated genes and the primer pairs for amplification can be found in Tables S5 (*B. napus*) and S6 (*A. thaliana*). Primers were designed to detect all *B. napus* homeologues when applicable and checked with Vector NTI (Thermo Fisher Scientific) for their properties. Primers were synthesized by MWG Eurofins and the annealing temperature was set according to the respective data sheets. Primer specificity was checked on the NCBI homepage (www.ncbi.nlm.nih.gov/tools/primer-blast/) by PRIMER BLAST analysis and subsequent to the qPCR, the determination of a melting curve of the amplified PCR products was carried out, followed by gel electrophoresis of the qPCR products to confirm single product amplification.

### Expression analysis by quantitative real‐time PCR

One microlitre of total RNA was treated with RNase‐free DNaseI (Fermentas) and then transcribed in a volume of 20 µL with the RevertAid First Strand cDNA Synthesis Kit (Thermo Scientific) into first‐strand cDNA according to supplier’s instructions. Two microlitres of a 1:10 diluted cDNA preparation was mixed with 18 µL Master Mix as described in the manual of the Maxima SYBR Green/ROX qPCR Master Mix (Thermo Scientific). PCR was performed on a CFX96 Touch Real‐Time PCR Detection System (Bio‐Rad) using the following conditions: 3 min at 95 °C; 45 cycles of 10 s at 95 °C, 10 s at 59 °C, 10 s at 72 °C; and 10 s at 95 °C, melting curve from 65 °C to 95 °C. Gene expression was determined using the ΔC_T_ method to calculate the relative expression according to Pfaffl (2001). Relative expression was calculated in relation to *PP2A* (At1G13320/BnaA06g33370D) and *Actin2* (At3G18780/BnaA10g22340D) as reference genes, which have been found to be stably expressed in many plants under almost all conditions (Czechowski *et al.*, 2005; Hruz *et al.*, 2008; Liu *et al.*, 2012) and with consideration of primer efficiency, which was estimated by standard curves for each gene generated by cDNA dilution series (1:10; 1:100; 1:1000). Each data point is based on three independent biological replicates measured with two technical replicates each. The relative expression to *PP2A*/*Actin2* was log_2_ transformed and, to display the differential regulation, the induction was calculated as the difference between *Vl43-* and mock‐treated samples. Error bars indicate the standard error of the mean and statistics were performed using Student’s *t*‐test (*P ≤ 0.05; **P ≤ 0.01; ***P ≤ 0.001), comparing the relative log_2_ expression between mock‐ and *Verticillium*‐treated samples of each calculated induction.

### ABA measurement from leaf and root material

Leaf and root tissue was groud under liquid nitrogen using mortar and pestle and 100 mg material was added to 1.5 mL 80% methanol and incubated overnight at 4 °C with shaking. Next day the 2 mL tubes were centrifuged for 10 min and 5000 rpm at 4 °C. One millilitre of the supernatant was transferred to a new tube and samples were dried in a speed vac until all liquid evaporated. The pellets were dissolved in 100 µL methanol by sonication for 15 min and afterwards 900 µL Tris‐buffered saline was added. One hundred microlitres of these samples was subjected to competitive ELISA from the Phytodetek^®^ ABA Test Kit (AGDIA, Elkhart, IN) according to the manufacturer’s instructions and measured at 405 nm with an ELISA reader Model 680 (Bio‐Rad). The amount of ABA was calculated as ng/100 mg FW and each data point was based on three independent biological replicates measured with two technical replicates each. Error bars indicate the standard error of the mean and statistics were performed using the Student’s *t*‐test (*P ≤ 0.05; **P ≤ 0.01; ***P ≤ 0.001) comparing mock‐ and *Vl43*‐treated samples, respectively.

### External application of ABA on oilseed rape seedlings

Seven‐day‐old oilseed rape seedlings were transferred on ½ MS medium without (mock) and with 10 µM ABA before inoculation. *S*eedlings were photographed at 6 dpi and hypocotyls were harvested for the quantification of fungal DNA as well as for *Vl43*‐hypocotyl assays.

### Genotyping of *A. thaliana* T‐DNA lines

T‐DNA knockout lines were ordered from NASC and were genotyped according to the instructions of the SALK Institute (http://signal.salk.edu/tdnaprimers.2.html; Table S7). The *nced3* knockout line has been selected on a T‐DNA insertion within exon 1, resulting in potential loss of function (At3G14440/GK‐129B08/N331021). The *aao3* knockout line was selected on a T‐DNA insertion within exon 3, resulting in potential loss of function (At2G27150/SALK_072361C/N668480).

### Statistical analyses

Graphs and plots were generated with Microsoft® Office 2007 and/or the ggplot2 package (Wickham, 2016) for R. All statistical calculations were performed with R (R core Team 2018). The comparison of means for two groups was performed using the Student’s or Welch *t*‐tests (*P ≤ 0.05; **P ≤ 0.01; ***P ≤ 0.001). Multiple comparison of means was performed by an analysis of variance (ANOVA) and a post hoc Tukey test (*P ≤ 0.05; **P ≤ 0.01; ***P ≤ 0.001) using SimComp (Hasler and Kluss, 2017).

## Supporting information


**Fig. S1** Scatterplot of all RNA‐Seq calculated FPKM values for all unigenes between mock‐ and *Vl43*‐infected samples for *Brassica napus* and *Arabidopsis thaliana*, respectively. Genes were considered to be differentially regulated when their log_2 _fold change was ≥1 (red) or ≤–1 (green).

**Fig. S2** ATTED‐II search for ABA‐related co‐expressed genes with the database of closely related model plant *Arabidopsis*. The identified coregulatory networks are shown (A–E) and have been partly merged together. Genes that have also been found in the RNA‐Seq dataset are highlighted in yellow. Genes identified in the networks A–D are down‐regulated in the RNA‐Seq data, while genes from cluster E are up‐regulated.

**Fig. S3** Characterization of the *in vitro *infection process of *Brassica napus *(Express 617) with *Verticillium longisporum (Vl43).* (A) Mock‐ and *Vl43*‐ inoculated *B. napus *seedlings were photographed at 3, 6 and 12 days post‐inoculation (dpi). (B) Hypocotyls were sampled and surface sterilized at different dpi and placed on potato dextrose agar to detect *Vl43 *in hypocotyls of infected *B. napus* seedlings. (C) Increment of fungal DNA in *B. napus *hypocotyls, measured by semiquantitative PCR using *Verticillium*‐ specific primers is displayed as the relative amount of fungal DNA at three different time points normalized by *PP2A* amplification from plant DNA. Error bars indicate the standard deviation (±SD) of three different biological repetitions and statistics were performed using the Student’s *t*‐test (*P ≤ 0.05).

**Fig. S4** Characterization of the *in vitro *infection process of *Arabidopsis thaliana *(Col‐0) with *Vertcillium longisporum (Vl43).* Mock‐ and *Vl43‐*inoculated Col‐0 were photographed at 3, 6, 9 and 12 days post‐infection (A) and detection of WGA‐FITC stained hyphae at 4 dpi was documented using confocal microscopy (B).

**Fig. S5** Disease symptom development in *Verticillium longisporum (Vl43‐*infected *Arabidopsis* and oilseed rape plants. Photos were taken at 36 days post‐inoculation.

**Fig. S6** Fungal detection in the leaves. Oilseed rape leaves were cut and surface sterilized at 12 days post‐inoculation (dpi) and subsequently placed on potato dextrose agar for 4 days. As can be seen, no fungal growth could be observed at 12 dpi, indicating fungal growth within the plant was still restricted to the hypocotyls.

**Fig. S7** Overview of the RNA‐Seq data procession pipeline for the *Brassica napus* experiment.

**Fig. S8** Effects of external application of abscisic acid (ABA) on the infection progression of *Verticillium longisporum* (*Vl43*) by oilseed rape seedlings. Seedlings were transferred on ½ MS medium without (mock) or with 10 µM ABA before inoculation. (A) *Brassica napus *seedlings were photographed at 6 days post‐inoculation (dpi). (B) Fungal DNA in hypocotyls was measured by semiquantitative PCR using *Verticillium*‐specific primers as described above. Error bars indicate the standard deviation (±SD) of three different biological repetitions, consisting of three plants each. (C) At 6 dpi mock‐ and ABA‐treated hypocotyls were surface sterilized, cut into three segments and placed on potato dextrose agar to observe fungal growth. For each treatment six plants were employed. Two weeks after hypocotyl transfer, colonized hypocotyls were counted. In the mock control five out of six hypocotyls were colonized, while after ABA treatment colonization was only observed in one case.

**Fig. S9** Differentially expressed genes related to abscisic acid (ABA) signalling as identified by RNA‐Seq of *Verticillium longisporum (Vl43)*‐infected oilseed rape (Bn) and *Arabidopsis* (At) roots at 6 days post‐inoculation. The line separates up‐and down‐regulated genes in response to the *Vl43* infection of *Brassica napus*. The meta‐analysis of the orthologous *Arabidopsis* counterparts in response to various abiotic stress treatments focuses on ABA (1: AT‐00110, 2: AT‐00420), high light (3: AT‐00246), cold (4: AT‐00640), drought (5: AT‐00560, 6: AT‐00419), heat (7: AT‐00387), osmotic (8: AT‐00120) and salt stress (9: AT‐00534) treatments.

**Table S1** Differentially expressed *Brassica napus* and *Arabidopsis thaliana* genes identified by RNA‐Seq.

**Table S2** GO annotation table containing GO terms, KEGG pathways and RNA‐Seq expression data for all genes for which homologues were identified between *Brassica napus* and *Arabidopsis thaliana.*

**Table S3** Complete list from the GO enrichment analysis of biological processes for *Brassica napus* and *Arabidopsis thaliana.*

**Table S4** Verification of RNA‐Seq data by qPCR (comparison of log_2_ values), *P* ≤ 0.05 (*), *P* ≤ 0.01 (**), *P* ≤ 0.001 (***).

**Table S5** *Brassica napus* primers for qPCR.

**Table S6** *Arabidopsis thaliana* primers for qPCR.

**Table S7** Primer used for *Arabidopsis* T‐DNA genotyping and fungal quantification.

## References

[mpp12867-bib-0001] Alexa, A. and Rahnenfuhrer, J. (2018) topGO: Enrichment Analysis for Gene Ontology. R package version 2.34.0. https://www.bioconductor.org/packages/release/bioc/html/topGO.html. Accessed 2 September 2019.

[mpp12867-bib-0003] Anderson, J.P. , Badruzsaufari, E. , Schenk, P.M. , Manners, J.M. , Desmond, O.J. , Ehlert, C. , Maclean, D.J. , Ebert, P.R. and Kazan, K. (2004) Antagonistic interaction between abscisic acid and jasmonate‐ethylene signaling pathways modulates defense gene expression and disease resistance in *Arabidopsis* . Plant Cell, 16, 3460–3479. 15548743 10.1105/tpc.104.025833 PMC535886

[mpp12867-bib-0004] Arc, E. , Sechet, J. , Corbineau, F. , Rajjou, L. and Marion‐Poll, A. (2013) ABA crosstalk with ethylene and nitric oxide in seed dormancy and germination. Front. Plant Sci. 4, 63. 23531630 10.3389/fpls.2013.00063 PMC3607800

[mpp12867-bib-0006] Cao, F.Y. , Yoshioka, K. and Desveaux, D. (2011) The roles of ABA in plant–pathogen interactions. J. Plant Res. 124, 489–499. 21380629 10.1007/s10265-011-0409-y

[mpp12867-bib-0007] Chalhoub, B. , Denoeud, F. , Liu, S. , Wincker, P. (2014) Plant genetics. Early allopolyploid evolution in the post‐Neolithic *Brassica napus* oilseed genome. Science, 345, 950–953. 25146293 10.1126/science.1253435

[mpp12867-bib-0008] Clay, N.K. , Adio, A.M. , Denoux, C. , Jander, G. and Ausubel, F.M. (2009) Glucosinolate metabolites required for an *Arabidopsis* innate immune response. Science, 323, 95–101. 19095898 10.1126/science.1164627 PMC2630859

[mpp12867-bib-0009] De Coninck, B. , Timmermans, P. , Vos, C. , Cammue, B.P. and Kazan, K. (2015) What lies beneath: belowground defense strategies in plants. Trends Plant Sci. 20, 91–101. 25307784 10.1016/j.tplants.2014.09.007

[mpp12867-bib-0011] Cui, F. , Brosché, M. , Lehtonen, M.T. , Amiryousefi, A. , Xu, E. , Punkkinen, M. , Valkonen, J.P. , Fujii, H. and Overmyer, K. (2016) Dissecting abscisic acid signaling pathways involved in cuticle formation. Mol. Plant, 9, 926–938. 27060495 10.1016/j.molp.2016.04.001

[mpp12867-bib-0012] Czechowski, T. , Stitt, M. , Altmann, T. , Udvardi, M.K. and Scheible, W.R. (2005) Genome‐wide identification and testing of superior reference genes for transcript normalization in *Arabidopsis* . Plant Physiol. 139, 5–17. 16166256 10.1104/pp.105.063743 PMC1203353

[mpp12867-bib-0013] Depotter, J.R. , Deketelaere, S. , Inderbitzin, P. , von Tiedemann, A. , Höfte, M. , Subbarao, K.V. , Wood, T.A. and Thomma, B.P. (2016) *Verticillium longisporum*, the invisible threat to oilseed rape and other Brassicaceous plant hosts. Mol. Plant Pathol. 17, 1004–1016. 26663851 10.1111/mpp.12350 PMC6638321

[mpp12867-bib-0014] Ellendorff, U. , Fradin, E.F. , de Jonge, R. and Thomma, B.P. (2009) RNA silencing is required for *Arabidopsis* defence against Verticillium wilt disease. J. Exp. Bot. 60, 591–602. 19098131 10.1093/jxb/ern306 PMC2651451

[mpp12867-bib-0015] Eynck, C. , Koopmann, B. , Grunewaldt‐Stoecker, G. , Karlovsky, P. and von Tiedemann, A. (2007) Differential interactions of *Verticillium longisporum* and *V. dahliae* with *Brassica napus* detected with molecular and histological techniques. Eur. J. Plant Pathol. 118, 259–274.

[mpp12867-bib-0016] Eynck, C. , Koopmann, B. and von Tiedemann, A. (2009) Identification of Brassica accessions with enhanced resistance to *Verticillium longisporum* under controlled and field conditions. J. Plant Dis. Protect. 116, 63–72.

[mpp12867-bib-0017] Fan, J. , Hill, L. , Crooks, C. , Doerner, P. and Lamb, C. (2009) Abscisic acid has a key role in modulating diverse plant–pathogen interactions. Plant Physiol. 150, 1750–1761. 19571312 10.1104/pp.109.137943 PMC2719142

[mpp12867-bib-0018] FAOSTAT . (2016) Statistical database. Food and Agriculture Organization of the United Nations: Statistic Division. Available at: http://www.fao.org/faostat/en/#data/QC/visualize. Accessed 9 December 2016.

[mpp12867-bib-0019] Finkelstein, R. (2013) Abscisic acid synthesis and response. Arabidopsis Book, 11, e0166. 24273463 10.1199/tab.0166 PMC3833200

[mpp12867-bib-0020] Floerl, S. , Druebert, C. , Majcherczyk, A. , Karlovsky, P. , Kües, U. and Polle, A. (2008) Defence reactions in the apoplastic proteome of oilseed rape (*Brassica napus* var. *napus*) attenuate *Verticillium longisporum* growth but not disease symptoms. BMC Plant Biol. 8, 129. 19094241 10.1186/1471-2229-8-129 PMC2644697

[mpp12867-bib-0021] Floerl, S. , Majcherczyk, A. , Possienke, M. , Feussner, K. , Tappe, H. , Gatz, C. , Feussner, I. , Kües, U. and Polle, A. (2012) *Verticillium longisporum* infection affects the leaf apoplastic proteome, metabolome, and cell wall properties in *Arabidopsis thaliana* . PLoS One, 7, e31435. 22363647 10.1371/journal.pone.0031435 PMC3282744

[mpp12867-bib-0022] Flors, V. , Ton, J. , van Doorn, R. , Jakab, G. , García‐Agustín, P. and Mauch‐Mani, B. (2008) Interplay between JA, SA and ABA signalling during basal and induced resistance against *Pseudomonas syringae* and *Alternaria brassicicola* . Plant J. 54, 81–92. 18088307 10.1111/j.1365-313X.2007.03397.x

[mpp12867-bib-0023] Fujita, M. , Fujita, Y. , Maruyama, K. , Seki, M. , Hiratsu, K. , Ohme‐Takagi, M. , Tran, L.S. , Yamaguchi‐Shinozaki, K. and Shinozaki, K. (2004) A dehydration‐induced NAC protein, RD26, is involved in a novel ABA‐dependent stress‐signaling pathway. Plant J. 39, 863–876. 15341629 10.1111/j.1365-313X.2004.02171.x

[mpp12867-bib-0025] Häffner, E. , Karlovsky, P. , Splivallo, R. , Traczewska, A. and Diederichsen, E. (2014) ERECTA, salicylic acid, abscisic acid, and jasmonic acid modulate quantitative disease resistance of *Arabidopsis thaliana* to *Verticillium longisporum* . BMC Plant Biol. 14, 85. 24690463 10.1186/1471-2229-14-85 PMC4021371

[mpp12867-bib-0026] Happstadius, I. , Ljunberg, A. , Kristiansson, B. and Dixelius, C. (2003) Identification of *Brassica oleracea* germplasm with improved resistance to Verticillium wilt. Plant Breed. 122, 30–34.

[mpp12867-bib-0044] Hasler, M. and Kluss, C. (2017) SimComp: Simultaneous Comparisons for Multiple Endpoints. R package version, 3.2. https://CRAN.R-project.org/package=SimComp. Accessed 2 September 2019.

[mpp12867-bib-0027] Hok, S. , Allasia, V. , Andrio, E. , Naessens, E. , Ribes, E. , Panabières, F. , Attard, A. , Ris, N. , Clément, M. , Barlet, X. and Marco, Y. (2014) The receptor kinase IMPAIRED OOMYCETE SUSCEPTIBILITY1 attenuates abscisic acid responses in *Arabidopsis* . Plant Physiol. 166, 1506–1518. 25274985 10.1104/pp.114.248518 PMC4226379

[mpp12867-bib-0028] Hruz, T. , Laule, O. , Szabo, G. , Wessendorp, F. , Bleuler, S. , Oertle, L. , Widmayer, P. , Gruissem, W. and Zimmermann, P. (2008) Genevestigator v3: a reference expression database for the meta‐analysis of transcriptomes. Adv. Bioinformatics, 2008, 420747. 10.1155/2008/420747 PMC2777001 19956698

[mpp12867-bib-0029] Inderbitzin, P. , Davis, R.M. , Bostock, R.M. and Subbarao, K.V. (2011) The ascomycete *Verticillium longisporum* is a hybrid and a plant pathogen with an expanded host range. PLoS One, 6, e18260. 21455321 10.1371/journal.pone.0018260 PMC3063834

[mpp12867-bib-0030] Iven, T. , König, S. , Singh, S. , Braus‐Stromeyer, S.A. , Bischoff, M. , Tietze, L.F. , Braus, G.H. , Lipka, V. , Feussner, I. and Dröge‐Laser, W. (2012) Transcriptional activation and production of tryptophan‐derived secondary metabolites in arabidopsis roots contributes to the defense against the fungal vascular pathogen *Verticillium longisporum* . Mol. Plant, 5, 1389–1402. 22522512 10.1093/mp/sss044

[mpp12867-bib-0031] Jensen, M.K. , Lindemose, S. , de Masi, F. , Reimer, J.J. , Nielsen, M. , Perera, V. , Workman, C.T. , Turck, F. , Grant, M.R. , Mundy, J. , Petersen, M. and Skriver, K. (2013) ATAF1 transcription factor directly regulates abscisic acid biosynthetic gene NCED3 in *Arabidopsis thaliana* . FEBS Open Bio. 3, 321–327. 10.1016/j.fob.2013.07.006 PMC3741915 23951554

[mpp12867-bib-0032] Jiang, Y. , Liang, G. and Yu, D. (2012) Activated expression of WRKY57 confers drought tolerance in *Arabidopsis* . Mol. Plant, 5, 1375–88. 22930734 10.1093/mp/sss080

[mpp12867-bib-0033] Johansson, A. , Staal, J. and Dixelius, C. (2006) Early responses in the *Arabidopsis*–*Verticillium longisporum* pathosystem are dependent on NDR1, JA‐ and ET‐associated signals via cytosolic NPR1 and RFO1. Mol. Plant‐Microbe Interact. 19, 958–969. 16941900 10.1094/MPMI-19-0958

[mpp12867-bib-0034] Kamble, A. , Koopmann, B. and von Tiedemann, A. (2013) Induced resistance to *Verticillium longisporum* in *Brassica napus* by β‐aminobutyric acid. Plant Pathol. 62, 552–561.

[mpp12867-bib-0036] Lamesch, P. , Berardini, T.Z. , Li, D. , Swarbreck, D. , Wilks, C. , Sasidharan, R. , Karthikeyan, A.S. (2011) The Arabidopsis Information Resource (TAIR): improved gene annotation and new tools. Nucleic Acids Res. 40(D1), D1202–D1210. 22140109 10.1093/nar/gkr1090 PMC3245047

[mpp12867-bib-0037] L'Haridon, F. , Besson‐Bard, A. , Binda, M. , Serrano, M. , Abou‐Mansour, E. , Balet, F. , Schoonbeek, H.J. , Hess, S. , Mir, R. , Léon, J. , Lamotte, O. and Métraux, J.P. (2011) A permeable cuticle is associated with the release of reactive oxygen species and induction of innate immunity. PLoS Pathog. 7, e1002148. 21829351 10.1371/journal.ppat.1002148 PMC3145797

[mpp12867-bib-0039] Liu, D. , Shi, L. , Han, C. , Yu, J. , Li, D. and Zhang, Y. (2012) Validation of reference genes for gene expression studies in virus‐infected *Nicotiana benthamiana* using quantitative real‐time PCR. PLoS One, 7, e46451. 23029521 10.1371/journal.pone.0046451 PMC3460881

[mpp12867-bib-0040] Liu, S. , Kracher, B. , Ziegler, J. , Birkenbihl, R.P. and Somssich, I.E. (2015a) Negative regulation of ABA signaling by WRKY33 is critical for *Arabidopsis* immunity towards *Botrytis cinerea* 2100. eLife, 4, e07295. 26076231 10.7554/eLife.07295 PMC4487144

[mpp12867-bib-0041] Liu, Z. , Yan, J.P. , Li, D.K. , Luo, Q. , Yan, Q. , Liu, Z.B. , Ye, L.M. , Wang, J.M. , Li, X.F. and Yang, Y. (2015b) UDP‐glucosyltransferase71c5, a major glucosyltransferase, mediates abscisic acid homeostasis in *Arabidopsis* . Plant Physiol. 167, 1659–1670. 25713337 10.1104/pp.15.00053 PMC4378179

[mpp12867-bib-0042] Lopisso, D.T. , Knüfer, J. , Koopmann, B. and von Tiedemann, A. (2017) The vascular pathogen *Verticillium longisporum* does not affect water relations and plant responses to drought stress of its host, *Brassica napus* . Phytopathology, 107, 444–454. 27992306 10.1094/PHYTO-07-16-0280-R

[mpp12867-bib-0043] Lorenzo, O. , Chico, J.M. , Sánchez‐Serrano, J.J. and Solano, R. (2004) JASMONATE‐INSENSITIVE1 encodes a MYC transcription factor essential to discriminate between different jasmonate‐regulated defense responses in *Arabidopsis* . Plant Cell, 16, 1938–1950. 15208388 10.1105/tpc.022319 PMC514172

[mpp12867-bib-0045] Mauch‐Mani, B. and Mauch, F. (2005) The role of abscisic acid in plant–pathogen interactions. Curr. Opin. Plant Biol. 8, 409–414. 15939661 10.1016/j.pbi.2005.05.015

[mpp12867-bib-0046] Mbengue, M. , Navaud, O. , Peyraud, R. , Barascud, M. , Badet, T. , Vincent, R. , Barbacci, A. and Raffaele, S. (2016) Emerging trends in molecular interactions between plants and the broad host range fungal pathogens *Botrytis cinerea* and *Sclerotinia sclerotiorum* . Front. Plant Sci. 31, 422. 10.3389/fpls.2016.00422 PMC4814483 27066056

[mpp12867-bib-0047] Mialoundama, A.S. , Heintz, D. , Debayle, D. , Rahier, A. , Camara, B. and Bouvier, F. (2009) Abscisic acid negatively regulates elicitor‐induced synthesis of capsidiol in wild tobacco. Plant Physiol. 150, 1556–1566. 19420326 10.1104/pp.109.138420 PMC2705044

[mpp12867-bib-0048] Morrison, E.N. , Emery, R.J. and Saville, B.J. (2015) Phytohormone involvement in the *Ustilago maydis*–*Zea mays* pathosystem: relationships between abscisic acid and cytokinin levels and strain virulence in infected cob tissue. PLoS One, 10, e0130945. 26107181 10.1371/journal.pone.0130945 PMC4479884

[mpp12867-bib-0049] Nakashima, K. , Yamaguchi‐Shinozaki, K. and Shinozaki, K. (2014) The transcriptional regulatory network in the drought response and its crosstalk in abiotic stress responses including drought, cold, and heat. Front. Plant Sci. 5, 170. 24904597 10.3389/fpls.2014.00170 PMC4032904

[mpp12867-bib-0050] Nishimura, N. , Okamoto, M. , Narusaka, M. , Yasuda, M. , Nakashita, H. , Shinozaki, K. , Narusaka, Y. and Hirayama, T. (2009) ABA hypersensitive germination2‐1 causes the activation of both abscisic acid and salicylic acid responses in *Arabidopsis* . Plant Cell Physiol. 50, 2112–2122. 19892832 10.1093/pcp/pcp146

[mpp12867-bib-0051] Obayashi, T. , Kinoshita, K. , Nakai, K. , Shibaoka, M. , Hayashi, S. , Saeki, M. , Shibata, D. , Saito, K. and Ohta, H. (2007). ATTED‐II: a database of co‐expressed genes and cis elements for identifying co‐regulated gene groups in Arabidopsis. Nucleic Acids Res. 35(Database issue), D863–9. 17130150 10.1093/nar/gkl783 PMC1716726

[mpp12867-bib-0052] Park, J. , Kim, Y.S. , Kim, S.G. , Jung, J.H. , Woo, J.C. and Park, C.M. (2011) Integration of auxin and salt signals by the NAC transcription factor NTM2 during seed germination in *Arabidopsis* . Plant Physiol. 156, 537–549. 21450938 10.1104/pp.111.177071 PMC3177257

[mpp12867-bib-0053] Pertea, M. , Kim, D. , Pertea, G.M. , Leek, J.T. and Salzberg, S.L. (2016) Transcript‐level expression analysis of RNA‐seq experiments with HISAT, StringTie and Ballgown. Nat. Protoc. 11, 1650. 27560171 10.1038/nprot.2016.095 PMC5032908

[mpp12867-bib-0054] Pfaffl, M.W. (2001) A new mathematical model for relative quantification in real‐time RT‐PCR. Nucleic Acids Res. 29, e45. 11328886 10.1093/nar/29.9.e45 PMC55695

[mpp12867-bib-0010] R Core Team . (2018) R: A Language and Environment for Statistical Computing. Vienna, Austria: R Foundation for Statistical Computing http://www.R-project.org/. Accessed 2 September 2019.

[mpp12867-bib-0055] Ralhan, A. , Schöttle, S. , Thurow, C. , Iven, T. , Feussner, I. , Polle, A. and Gatz, C. (2012) The vascular pathogen *Verticillium longisporum* requires a jasmonic acid‐independent COI1 function in roots to elicit disease symptoms in *Arabidopsis* shoots. Plant Physiol. 159, 1192–1203. 22635114 10.1104/pp.112.198598 PMC3387704

[mpp12867-bib-0056] Ratzinger, A. , Riediger, N. , von Tiedemann, A. and Karlovsky, P. (2009) Salicylic acid and salicylic acid glucoside in xylem sap of *Brassica napus* infected with *Verticillium longisporum* . J Plant Res. 122, 571–579. 19449088 10.1007/s10265-009-0237-5 PMC2776162

[mpp12867-bib-0057] Reusche, M. , Thole, K. , Janz, D. , Truskina, J. , Rindfleisch, S. , Drübert, C. , Polle, A. , Lipka, V. and Teichmann, T. (2012) *Verticillium* infection triggers VASCULAR‐RELATED NAC DOMAIN7‐dependent *de novo* xylem formation and enhances drought tolerance in *Arabidopsis* . Plant Cell, 24, 3823–3837. 23023171 10.1105/tpc.112.103374 PMC3480305

[mpp12867-bib-0058] Reusche, M. , Truskina, J. , Thole, K. , Nagel, L. , Rindfleisch, S. , Tran, V.T. , Braus‐Stromeyer, S.A. , Braus, G.H. , Teichmann, T. and Lipka, V. (2014) Infections with the vascular pathogens *Verticillium longisporum* and *Verticillium dahliae* induce distinct disease symptoms and differentially affect drought stress tolerance of *Arabidopsis thaliana* . Environ. Exp. Bot. 108, 23–37.

[mpp12867-bib-0059] Rogers, S.O. and Bendich, A.J. (1985) Extraction of DNA from milligram amounts of fresh, herbarium and mummified plant tissues. Plant Mol. Biol. 5, 69–76. 24306565 10.1007/BF00020088

[mpp12867-bib-0060] Roos, J. , Bejai, S. , Oide, S. and Dixelius, C. (2014) RabGAP22 is required for defense to the vascular pathogen *Verticillium longisporum* and contributes to stomata immunity. PLoS One, 9, e88187. 24505423 10.1371/journal.pone.0088187 PMC3913773

[mpp12867-bib-0061] Roos, J. , Bejai, S. , Mozūraitis, R. and Dixelius, C. (2015) Susceptibility to *Verticillium longisporum* is linked to monoterpene production by TPS23/27 in *Arabidopsis* . Plant J. 81, 572–85. 25640950 10.1111/tpj.12752

[mpp12867-bib-0062] Rushton, D.L. , Tripathi, P. , Rabara, R.C. , Lin, J. , Ringler, P. , Boken, A.K. , Langum, T.J. , Smidt, L. , Boomsma, D.D. , Emme, N.J. , Chen, X. , Finer, J.J. , Shen, Q.J. and Rushton, P.J. (2012) WRKY transcription factors: key components in abscisic acid signalling. Plant Biotechnol. J. 10, 2. 21696534 10.1111/j.1467-7652.2011.00634.x

[mpp12867-bib-0063] Rygulla, W. , Friedt, W. , Seyis, F. , Lühs, W. , Eynck, C. , von Tiedemann, A. and Snowdon, R.J. (2007a) Combination of resistance to *Verticillium longisporum* from zero erucic acid *Brassica oleracea* and oilseed *Brassica rapa* genotypes in resynthesized rapeseed (*Brassica napus*) lines. Plant Breed. 126, 596–602.

[mpp12867-bib-0064] Rygulla, W. , Snowdon, R.J. , Eynck, C. , Koopmann, B. , Von Tiedemann, A. , Lühs, W. and Friedt, W. (2007b) Broadening the genetic basis of *Verticillium longisporum* resistance in *Brassica napus* by interspecific hybridisation. Phytopathology, 97, 1391–1396. 18943507 10.1094/PHYTO-97-11-1391

[mpp12867-bib-0065] Sánchez‐Vallet, A. , López, G. , Ramos, B. , Delgado‐Cerezo, M. , Riviere, M.P. , Llorente, F. , Fernández, P.V. , Miedes, E. , Estevez, J.M. , Grant, M. and Molina, A. (2012) Disruption of abscisic acid signaling constitutively activates *Arabidopsis* resistance to the necrotrophic fungus *Plectosphaerella cucumerina* . Plant Physiol. 160, 2109–2124. 23037505 10.1104/pp.112.200154 PMC3510135

[mpp12867-bib-0066] Shang, Y. , Yan, L. , Liu, Z.Q. , Cao, Z. , Mei, C. , Xin, Q. , Wu, F.Q. , Wang, X.F. , Du, S.Y. , Jiang, T. , Zhang, X.F. , Zhao, R. , Sun, H.L. , Liu, R. , Yu, Y.T. and Zhang, D.P. (2010) The Mg‐chelatase H subunit of *Arabidopsis* antagonizes a group of WRKY transcription repressors to relieve ABA‐responsive genes of inhibition. Plant Cell, 22, 1909–1935. 20543028 10.1105/tpc.110.073874 PMC2910980

[mpp12867-bib-0067] Shen, D. , Suhrkamp, I. , Wang, Y. , Liu, S. , Menkhaus, J. , Verreet, J.A. , Fan, L.J. and Cai, D. (2014) Identification and characterization of microRNAs in oilseed rape (*Brassica napus*) responsive to infection with the pathogenic fungus *Verticillium longisporum* using Brassica AA (*Brassica rapa*) and CC (*Brassica oleracea*) as reference genomes. New Phytol. 204, 577–594. 25132374 10.1111/nph.12934

[mpp12867-bib-0068] Siewers, V. , Kokkelink, L. , Smedsgaard, J. and Tudzynski, P. (2006) Identification of an abscisic acid gene cluster in the grey mold *Botrytis cinerea* . Appl. Environ. Microbiol. 72, 4619–4626. 16820452 10.1128/AEM.02919-05 PMC1489360

[mpp12867-bib-0069] Spence, C.A. , Lakshmanan, V. , Donofrio, N. and Bais, H.P. (2015) Crucial roles of abscisic acid biogenesis in virulence of rice blast fungus *Magnaporthe oryzae* . Front. Plant Sci. 6, 1082. 26648962 10.3389/fpls.2015.01082 PMC4664623

[mpp12867-bib-0070] Sun, F. , Fan, G. , Hu, Q. , Zhou, Y. , Guan, M. , Wang, H. (2017) The high‐quality genome of *Brassica napus* cultivar 'ZS11' reveals the introgression history in semi‐winter morphotype. Plant J. 92, 452–468. 28849613 10.1111/tpj.13669

[mpp12867-bib-0071] Thaler, J.S. , Humphrey, P.T. and Whiteman, N.K. (2012) Evolution of jasmonate and salicylate signal crosstalk. Trends Plant Sci. 17, 260–270. 22498450 10.1016/j.tplants.2012.02.010

[mpp12867-bib-0072] Tian, H. , Guo, H. , Dai, X. , Cheng, Y. , Zheng, K. , Wang, X. and Wang, S. (2015) An ABA down‐regulated bHLH transcription repressor gene, *bHLH129* regulates root elongation and ABA response when overexpressed in *Arabidopsis* . Sci. Rep. 5, 17587. 26625868 10.1038/srep17587 PMC4667245

[mpp12867-bib-0073] Ton, J. , Flors, V. and Mauch‐Mani, B. (2009) The multifaceted role of ABA in disease resistance. Trends Plant Sci. 14, 310–317. 19443266 10.1016/j.tplants.2009.03.006

[mpp12867-bib-0076] de Torres‐Zabala, M. , Truman, W. , Bennett, M.H. , Lafforgue, G. , Mansfield, J.W. , Rodriguez Egea, P. , Bögre, L. and Grant, M. (2007) *Pseudomonas syringae* pv. *tomato* hijacks the *Arabidopsis* abscisic acid signaling pathway to cause disease. EMBO J. 26, 1434–1443. 17304219 10.1038/sj.emboj.7601575 PMC1817624

[mpp12867-bib-0075] de Torres-Zabala, M. , Bennett, M.H. , Truman, W.H. and Grant, M.R. (2009) Antagonism between salicylic and abscisic acid reflects early host‐pathogen conflict and moulds plant defence responses. Plant J. 59, 375–386. 19392690 10.1111/j.1365-313X.2009.03875.x

[mpp12867-bib-0074] de Torres-Zabala M. , Littlejohn, G. , Jayaraman, S. , Studholme, D. , Bailey, T. , Lawson, T. , Tillich, M. , Licht, D. , Bölter, B. , Delfino, L. , Truman, W. , Mansfield, J. , Smirnoff, N. and Grant, M. (2015) Chloroplasts play a central role in plant defence and are targeted by pathogen effectors. Nat. Plants, 1, 15074. 27250009 10.1038/nplants.2015.74

[mpp12867-bib-0077] Veronese, P. , Narasimhan, M.L. , Stevenson, R.A. , Zhu, J.K. , Weller, S.C. , Subbarao, K.V. and Bressan, R.A. (2003) Identification of a locus controlling *Verticillium* disease symptom response in *Arabidopsis thaliana* . Plant J. 35, 574–587. 12940951 10.1046/j.1365-313x.2003.01830.x

[mpp12867-bib-0078] Wang, M. , Weiberg, A. , Lin, F.M. , Thomma, B.P. , Huang, H.D. and Jin, H. (2016) Bidirectional cross‐kingdom RNAi and fungal uptake of external RNAs confer plant protection. Nat. Plants, 2, 16151. 27643635 10.1038/nplants.2016.151 PMC5040644

[mpp12867-bib-0079] Wickham, H. (2016) ggplot2: Elegant Graphics for Data Analysis. New York: Springer‐Verlag.

[mpp12867-bib-0080] Wu, J. , Mao, X. , Cai, T. , Luo, J. and Wei, L. (2006) KOBAS server: a web‐based platform for automated annotation and pathway identification. Nucleic Acids Res. 34, W720–W724. 16845106 10.1093/nar/gkl167 PMC1538915

[mpp12867-bib-0081] Xu, X. , Chen, C. , Fan, B. and Chen, Z. (2006) Physical and functional interactions between pathogen‐induced *Arabidopsis* WRKY18, WRKY40 and WRKY60 transcription factors. Plant Cell, 18, 1310–1326. 16603654 10.1105/tpc.105.037523 PMC1456877

[mpp12867-bib-0082] Xu, J. , Audenaert, K. , Hofte, M. and De Vleesschauwer, D. (2013) Abscisic acid promotes susceptibility to the rice leaf blight pathogen *Xanthomonas oryzae* pv. *oryzae* by suppressing salicylic acid‐mediated defenses. PLoS One, 8, e67413. 23826294 10.1371/journal.pone.0067413 PMC3694875

[mpp12867-bib-0083] Yasuda, M. , Ishikawa, A. , Jikumaru, Y. , Seki, M. , Umezawa, T. , Asami, T. , Maruyama‐Nakashita, A. , Kudo, T. , Shinozaki, K. , Yoshida, S. and Nakashita, H. (2008) Antagonistic interaction between systemic acquired resistance and the abscisic acid‐mediated abiotic stress response in *Arabidopsis* . Plant Cell, 20, 1678–1692. 18586869 10.1105/tpc.107.054296 PMC2483369

[mpp12867-bib-0084] Yoshida, T. , Mogami, J. and Yamaguchi‐Shinozaki, K. (2014) ABA‐dependent and ABA‐independent signaling in response to osmotic stress in plants. Curr. Opin. Plant Biol. 21, 133–139. 25104049 10.1016/j.pbi.2014.07.009

[mpp12867-bib-0085] Zhang, T. , Zhao, Y.L. , Zhao, J.H. , Wang, S. , Jin, Y. , Chen, Z.Q. , Fang, Y.Y. , Hua, C.L. , Ding, S.W. and Guo, H.S. (2016) Cotton plants export microRNAs to inhibit virulence gene expression in a fungal pathogen. Nat. Plants, 2, 16153. 27668926 10.1038/nplants.2016.153

