# Supplementary figures and images for: Suppression of abscisic acid biosynthesis at the early infection stage of Verticillium longisporum in oilseed rape (Brassica napus)
Source: Mol Plant Pathol. 2019 Oct 11;20(12):1645–61. doi: 10.1111/mpp.12867 (PMC6859492; doi:10.1111/mpp.12867)

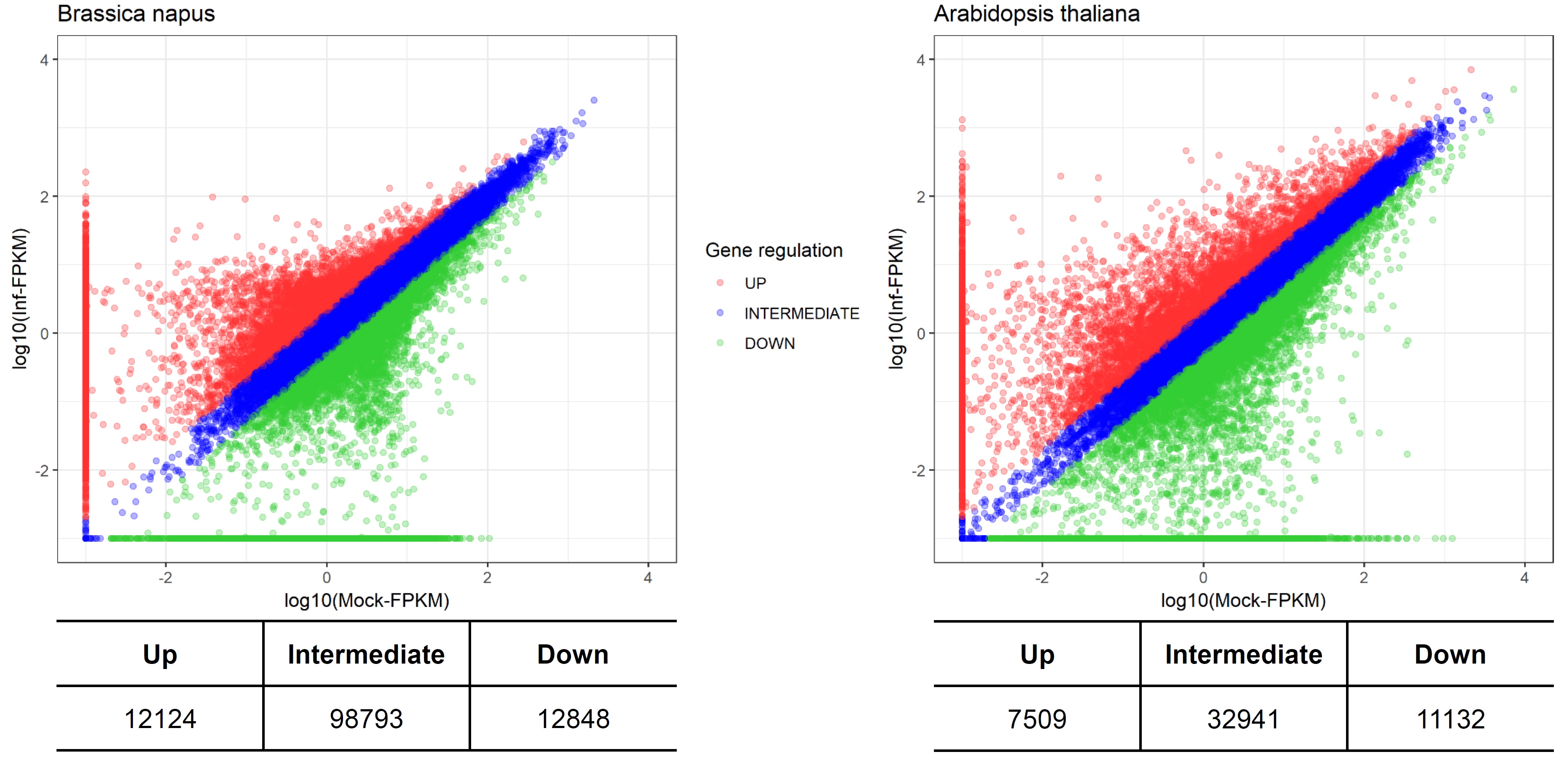

Supplement: Supplementary file 1 — Fig. S1 Scatterplot of all RNA‐Seq calculated FPKM values for all unigenes between mock‐ and Vl43‐infected samples for Brassica napus and Arabidopsis thaliana, respectively. Genes were considered to be differentially regulated when their log2 fold change was ≥1 (red) or ≤–1 (green). [file MPP-20-1645-s001.jpg]

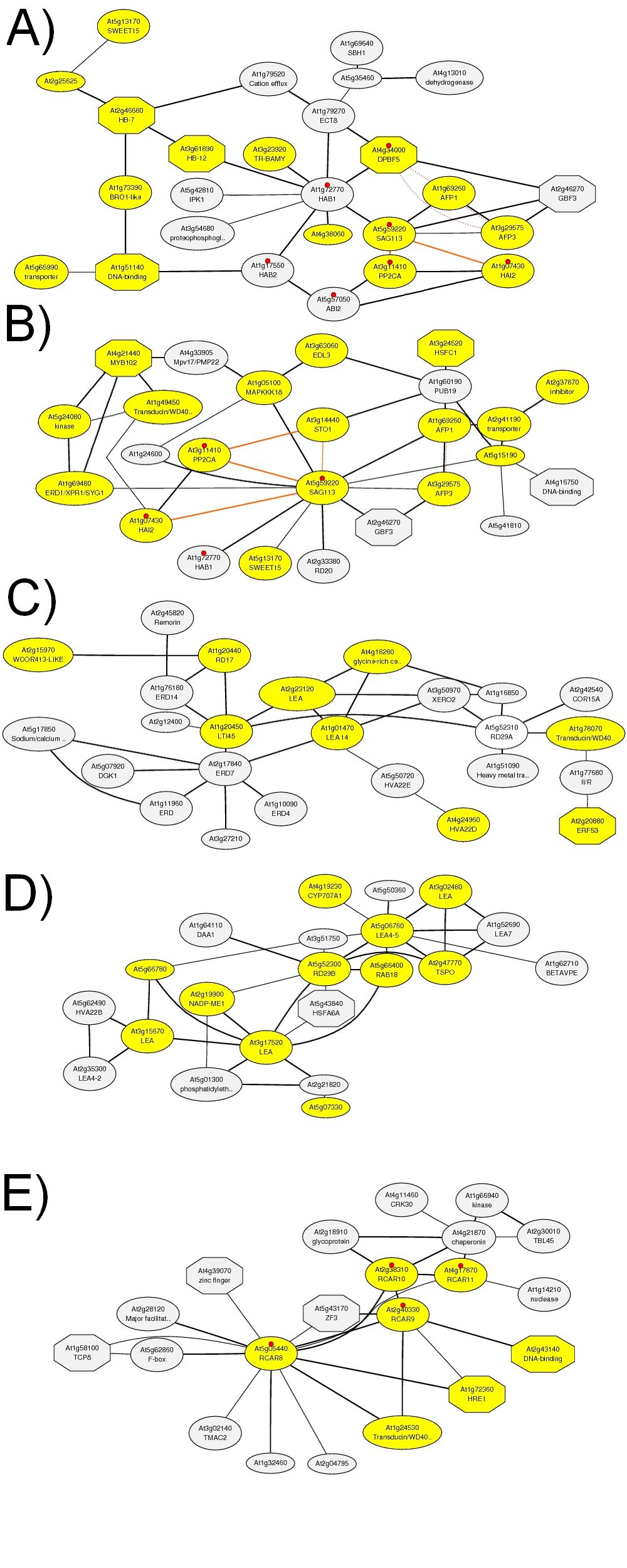

Supplement: Supplementary file 2 — Fig. S2 ATTED‐II search for ABA‐related co‐expressed genes with the database of closely related model plant Arabidopsis. The identified coregulatory networks are shown (A–E) and have been partly merged together. Genes that have also been found in the RNA‐Seq dataset are highlighted in yellow. Genes identified in the networks A–D are down‐regulated in the RNA‐Seq data, while genes from cluster E are up‐regulated. [file MPP-20-1645-s002.jpg]

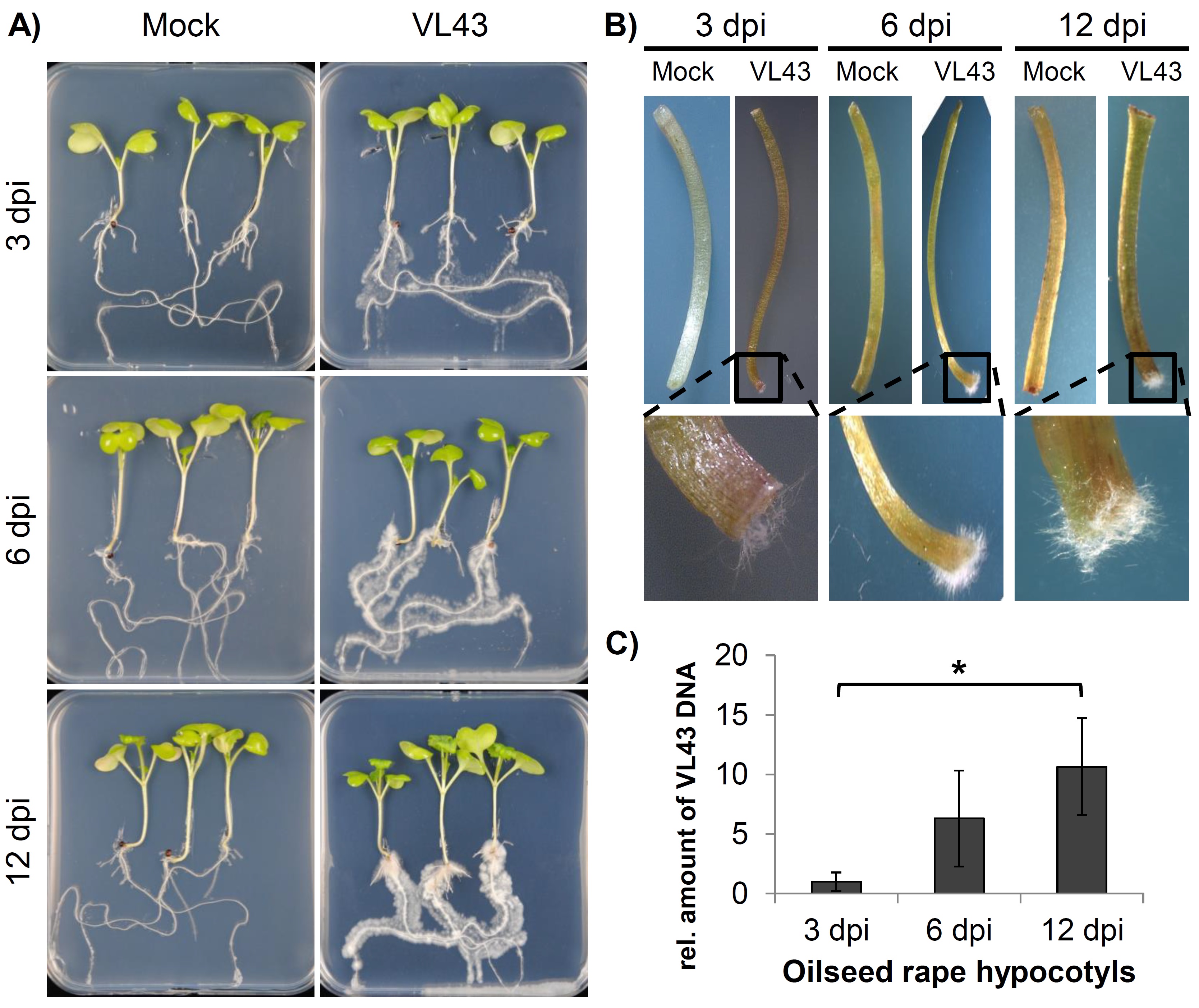

Supplement: Supplementary file 3 — Fig. S3 Characterization of the in vitro infection process of Brassica napus (Express 617) with Verticillium longisporum (Vl43). (A) Mock‐ and Vl43‐ inoculated B. napus seedlings were photographed at 3, 6 and 12 days post‐inoculation (dpi). (B) Hypocotyls were sampled and surface sterilized at different dpi and placed on potato dextrose agar to detect Vl43 in hypocotyls of infected B. napus seedlings. (C) Increment of fungal DNA in B. napus hypocotyls, measured by semiquantitative PCR using Verticillium‐ specific primers is displayed as the relative amount of fungal DNA at three different time points normalized by PP2A amplification from plant DNA. Error bars indicate the standard deviation (±SD) of three different biological repetitions and statistics were performed using the Student’s t‐test (*P ≤ 0.05). [file MPP-20-1645-s003.jpg]

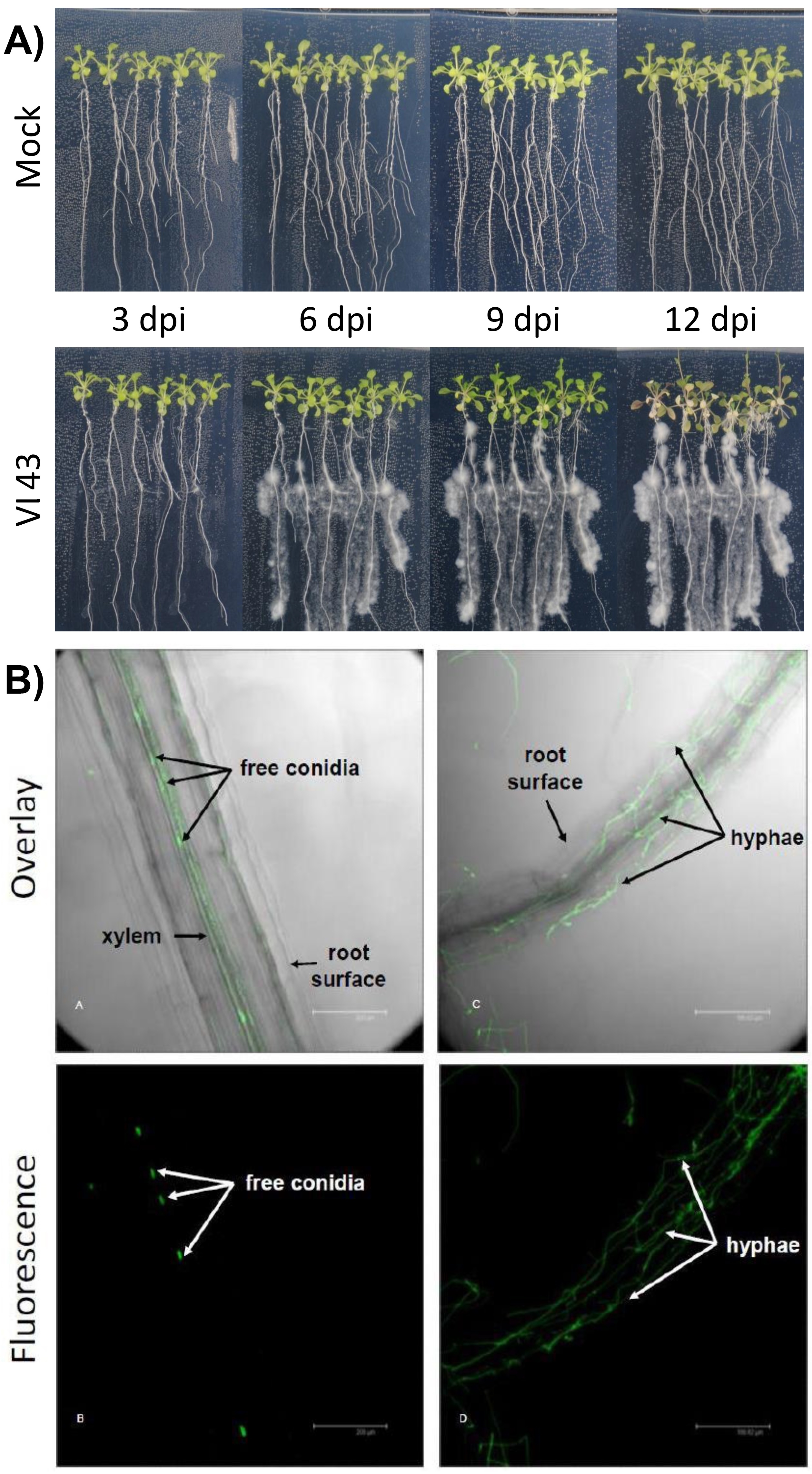

Supplement: Supplementary file 4 — Fig. S4 Characterization of the in vitro infection process of Arabidopsis thaliana (Col‐0) with Vertcillium longisporum (Vl43). Mock‐ and Vl43‐inoculated Col‐0 were photographed at 3, 6, 9 and 12 days post‐infection (A) and detection of WGA‐FITC stained hyphae at 4 dpi was documented using confocal microscopy (B). [file MPP-20-1645-s004.jpg]

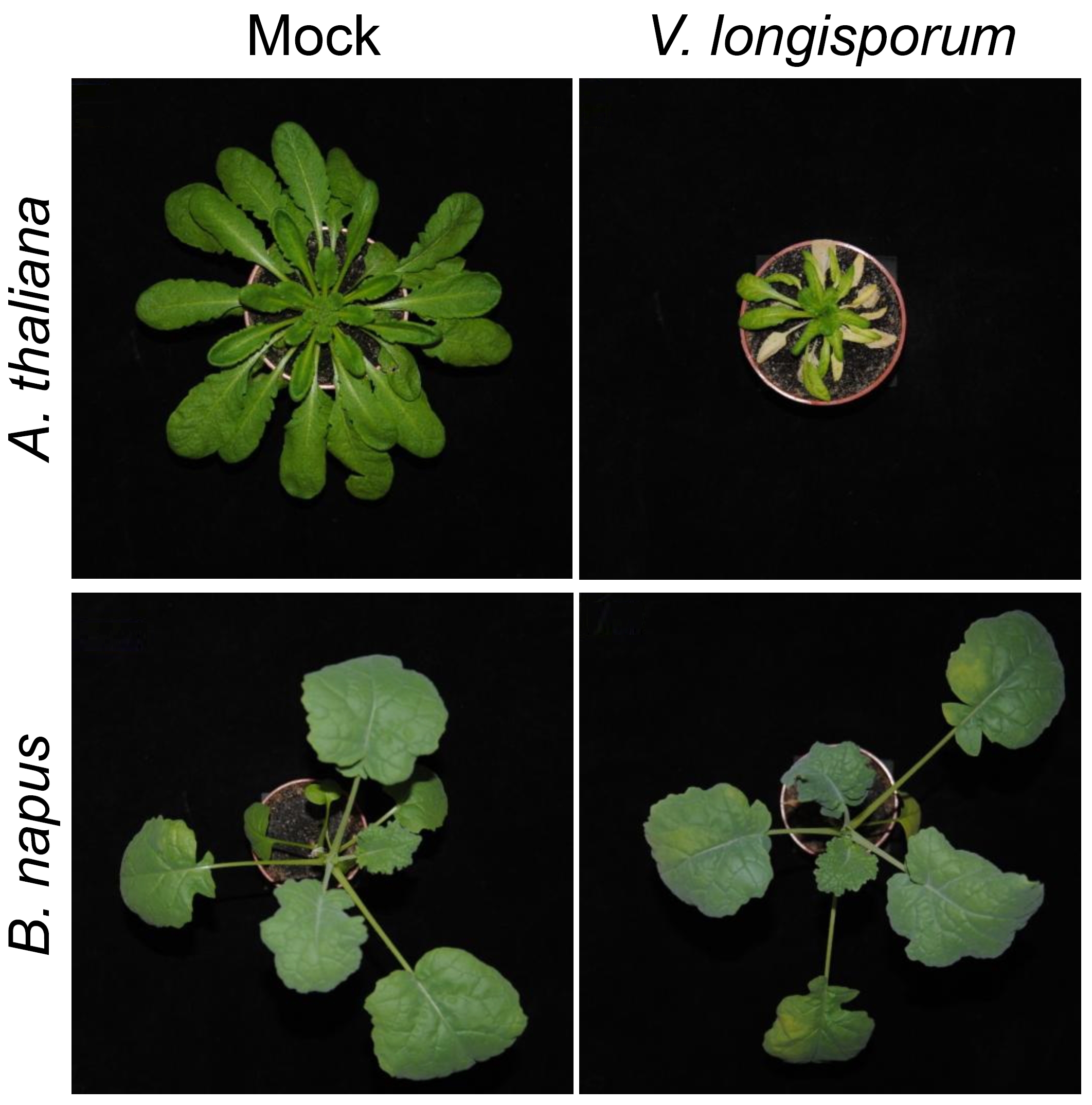

Supplement: Supplementary file 5 — Fig. S5 Disease symptom development in Verticillium longisporum (Vl43‐infected Arabidopsis and oilseed rape plants. Photos were taken at 36 days post‐inoculation. [file MPP-20-1645-s005.jpg]

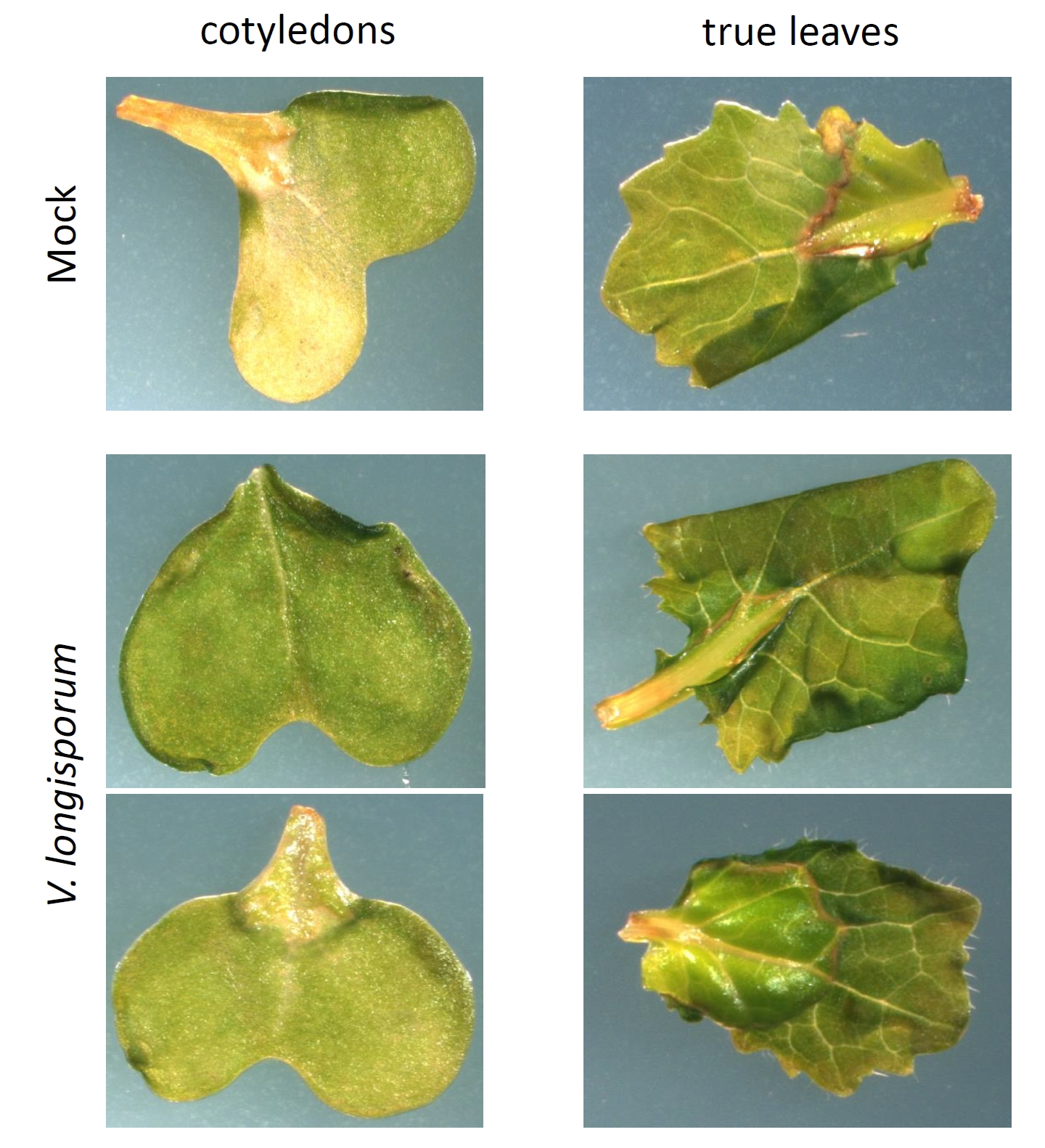

Supplement: Supplementary file 6 — Fig. S6 Fungal detection in the leaves. Oilseed rape leaves were cut and surface sterilized at 12 days post‐inoculation (dpi) and subsequently placed on potato dextrose agar for 4 days. As can be seen, no fungal growth could be observed at 12 dpi, indicating fungal growth within the plant was still restricted to the hypocotyls. [file MPP-20-1645-s006.jpg]

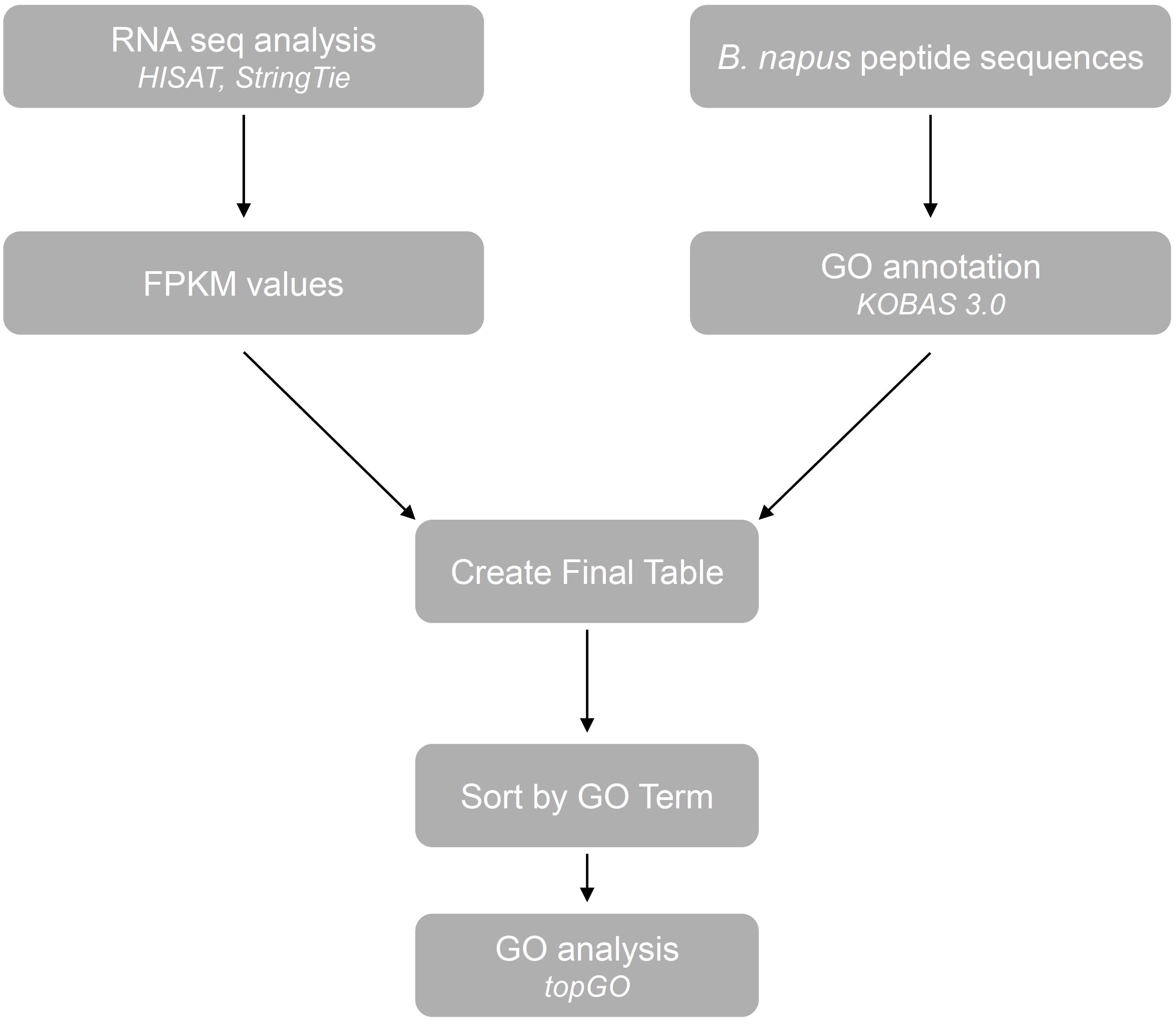

Supplement: Supplementary file 7 — Fig. S7 Overview of the RNA‐Seq data procession pipeline for the Brassica napus experiment. [file MPP-20-1645-s007.jpg]

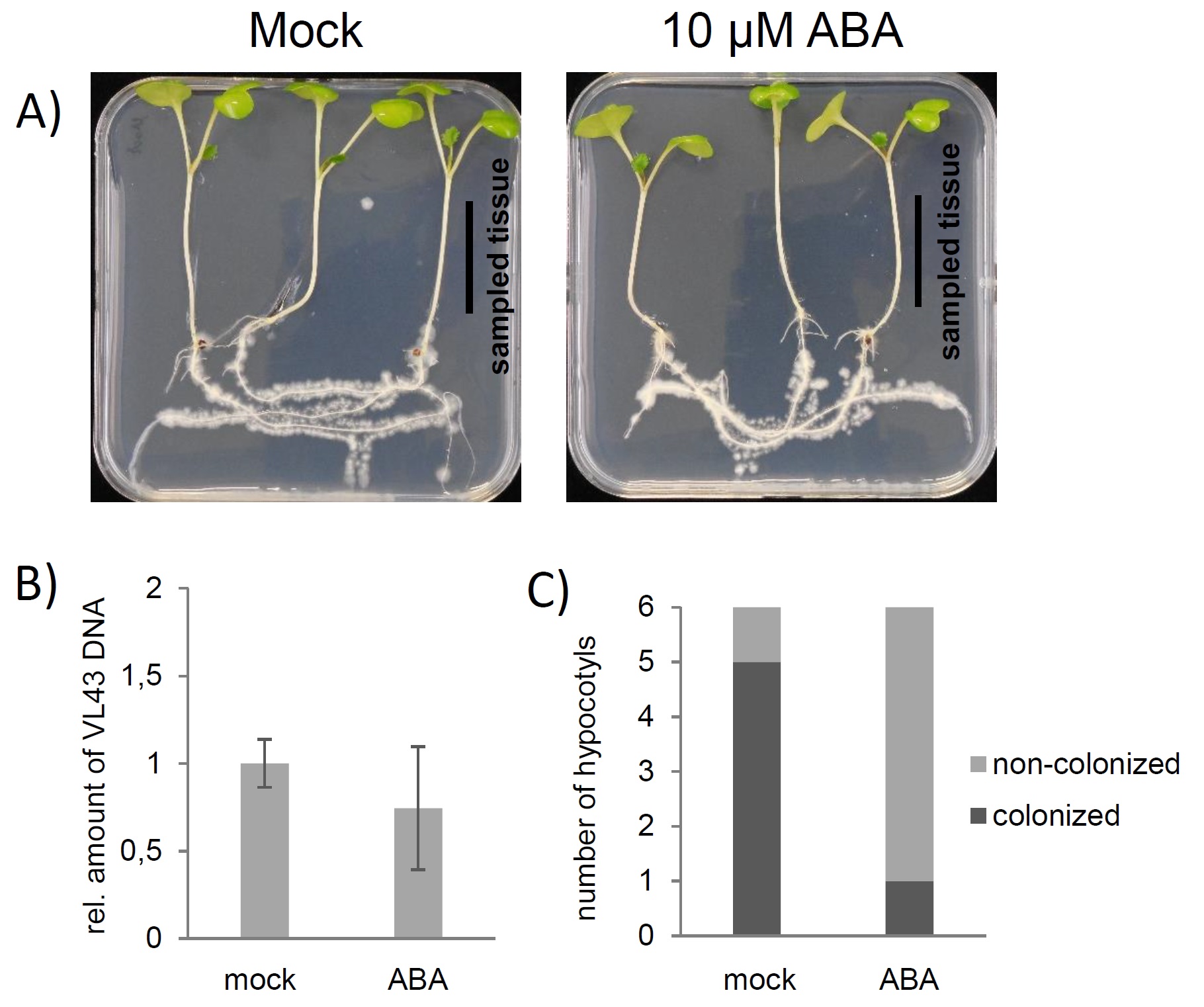

Supplement: Supplementary file 8 — Fig. S8 Effects of external application of abscisic acid (ABA) on the infection progression of Verticillium longisporum (Vl43) by oilseed rape seedlings. Seedlings were transferred on ½ MS medium without (mock) or with 10 µM ABA before inoculation. (A) Brassica napus seedlings were photographed at 6 days post‐inoculation (dpi). (B) Fungal DNA in hypocotyls was measured by semiquantitative PCR using Verticillium‐specific primers as described above. Error bars indicate the standard deviation (±SD) of three different biological repetitions, consisting of three plants each. (C) At 6 dpi mock‐ and ABA‐treated hypocotyls were surface sterilized, cut into three segments and placed on potato dextrose agar to observe fungal growth. For each treatment six plants were employed. Two weeks after hypocotyl transfer, colonized hypocotyls were counted. In the mock control five out of six hypocotyls were colonized, while after ABA treatment colonization was only observed in one case. [file MPP-20-1645-s008.jpg]
