# Supplementary material for: Suppression of abscisic acid biosynthesis at the early infection stage of Verticillium longisporum in oilseed rape (Brassica napus)
Source: Mol Plant Pathol. 2019 Oct 11;20(12):1645–61. doi: 10.1111/mpp.12867 (PMC6859492; doi:10.1111/mpp.12867)

Differentially expressed gene related to ABA signaling	Bn vs Vl at 6dpi		At vs Vl at 6dpi	
**Gene description**	**Bn ID**	**log2**	**At AGI**	**log2**
ERF073, HRE1 (HYPOXIA RESPONSIVE ERF1)	BnaA07g30130D	11.663	AT1G72360	-0.5415
ERF/AP2 TF, function as ABA REPRESSOR1 (ABR1)	BnaC03g49530D	1.5811	AT5G64750	5.1186
MATE efflux family protein, ABA transport	BnaC02g15390D	3.4694	AT5G52050	5.4013
abscisic acid receptor PYL6	BnaA04g29300D	3.3617	AT2G40330	1.5988
WRKY18	BnaAnng32610D	2.0336	AT4G31800	2.8484
bHLH129 TF	BnaC04g02750D	2.1991	AT2G43140	-3.3770
WRKY60	BnaC01g06660D	10.870	AT2G25000	2.8484
ARCK1, negatively controls abscisic acid signal transduction	BnaC09g23650D	3.1568	AT4G11890	4.9781
abscisic acid receptor PYL4	BnaA03g17720D	1.8967	AT2G38310	0.3351
abscisic acid 8'-hydroxylase 3 / CYP707A3	BnaCnng57040D	1.9445	AT5G45340	-1.1718
PYL5, PYRABACTIN RESISTANCE 1-LIKE 5, RCAR8	BnaC09g49910D	1.7998	AT5G05440	0.1279
SNF1-RELATED KINASE, SRK2H	BnaC03g50700D	0.7669	AT5G63650	-1.8615
PYR1, PYRABACTIN RESISTANCE 1, RCAR11	BnaCnng65400D	2.1669	AT4G17870	-1.3852
NAC69	BnaCnng01510D	1.1650	AT4G01550	-0.0535
Transducin/WD40 repeat-like superfamily protein	BnaC05g21010D	1.0445	AT1G24530	-1.2293
ABI5 BINDING PROTEIN 4, AFP4, negative regulator of ABA responses	BnaA03g27740D	0.7709	AT3G02140	-0.3041
UGT71C5, plays a role in abscisic acid (ABA) glucosylation	BnaA10g04700D	0.7307	AT1G07240	0.9366
WRKY40	BnaC06g40170D	1.0274	AT1G80840	3.9006
WRKY33	BnaC03g21360D	1.1279	AT2G38470	1.7308
SAD2, Regulates microRNA activity	BnaC03g17650D	10.539	AT2G31660	-0.9900
ATAF1, NAC TF that attentuates ABA signaling and synthesis	BnaA10g00280D	-0.3392	AT1G01720	1.2109
AAO3, ABA synthesis	BnaC04g38650D	-1.3790	AT2G27150	-0.8100
SNF1-RELATED KINASE, SRK2B	BnaA01g23120D	-0.6533	AT1G60940	1.2300
ABF4, bZIP transcription factor with specificity for ABRE	BnaA01g26200D	-6.3684	AT3G19290	-0.3801
ABI2, PP2C	BnaA10g11080D	-0.6007	AT5G57050	0.4461
beta-amylase 1	BnaC09g21440D	-1.0741	AT3G23920	0.3131
Endosomal targeting BRO1-like domain-containing protein	BnaA07g30840D	-1.6760	AT1G73390	-0.4456
WRKY57	BnaC06g25390D	-1.3657	AT1G69310	0.2194
ABF3.1 / abscisic acid responsive elements-binding factor 3	BnaC07g44670D	-1.7894	AT4G34000	-0.6276
bHLH122 TF / ABA-RESPONSIVE KINASE SUBSTRATE 1	BnaC06g04380D	-1.0263	AT1G51140	-0.7522
transmembrane amino acid transporter	BnaAnng06860D	-1.1192	AT5G65990	3.8039
Transducin/WD40 repeat-like superfamily protein	BnaA07g33920D	-0.7864	AT1G78070	0.3227
homeobox-leucine zipper protein ATHB-7 / ABA responsive	BnaC04g00750D	-1.3180	AT2G46680	1.3826
Late embryogenesis abundant protein, group 6	BnaC08g34610D	-1.3383	AT2G23120	-0.2492
uncharacterized hypothetical protein	BnaA08g15980D	-1.4000	AT4G38060	0.4345
ABA insensitive PP2C protein 1 (ABI1.1)	BnaC01g18020D	-1.1515	AT4G26080	0.2282
BG1, hydrolysis of Glc-conjugated ABA	BnaA06g01540D	-1.1853	AT1G52400	1.3261
HSFC1, HEAT SHOCK TRANSCRIPTION FACTOR C1	BnaA03g37460D	-1.4203	AT3G24520	-1.8831
cold regulated 413 plasma membrane 1 / Cyclophillin 19	BnaA07g03740D	-1.4441	AT2G15970	-0.9646
TSPO, (OUTER MEMBRANE TRYPTOPHAN-RICH SENSORY PROTEIN)	BnaA04g29550D	-1.5296	AT2G47770	3.3678
homeobox-leucine zipper protein ATHB-12	BnaCnng73330D	-1.6374	AT3G61890	1.3897
ABA-insensitive 5-like / ABA responsive elements-binding factor 2	BnaA10g28780D	-2.4677	AT1G45249	-0.7033
AFP2, ABI5 BINDING PROTEIN 2	BnaA09g46130D	-1.3810	AT1G13740	0.1091
abscisic acid 8'-hydroxylase 1 / CYP707A1	BnaC07g35800D	-2.0872	AT4G19230	0.2051
Ninja-family protein AFP1 (ABI FIVE BINDING PROTEIN) ABA / JA	BnaC06g25430D	-1.7578	AT1G69260	0.7057
NADP-MALIC ENZYME 1, ATNADP-ME1, NADP-MALIC ENZYME 1	BnaA07g00860D	-2.1470	AT2G19900	1.0571
Dehydrin, cold regulated / ERD10	BnaA07g11450D	-2.0560	AT1G20450	-0.2714
Caleosin-related family protein, neg. regulator in ABA signaling	BnaA02g15090D	-1.4054	AT1G70670	0.0377
abscisic acid-induced-like protein / Hva22D	BnaA03g47030D	-1.8737	AT4G24960	0.5657
nodulin MtN3 family protein / sugar transporter SWEET15-like / SAG29	BnaC02g04530D	-2.0040	AT5G13170	1.5888
G-type lectin S-receptor-like serine/threonine-protein kinase	BnaC07g30350D	-2.0165	AT5G24080	8.5085
NAC92	BnaC04g31690D	-1.3042	AT5G39610	1.0904
EID1-like F-box protein 3	BnaC04g09800D	-2.0631	AT3G63060	0.6533
MYB121	BnaA06g30710D	-2.1413	AT3G30210	2.1432
nodulin MtN3 family protein / SWEET1	BnaA06g15180D	-2.3379	AT1G21460	1.2727
HAI3, highly ABA-induced PP2C protein 3	BnaC04g14090D	-2.4450	AT2G29380	5.7099
NAC72, RD26	BnaC07g40860D	-2.3240	AT4G27410	-1.3826
vacuolar amino acid transporter 1-like	BnaA05g02050D	-3.3347	AT2G41190	-0.1871
DREB2A	BnaC02g02300D	-1.2658	AT5G05410	2.3515
late embryogenesis abundant protein	BnaCnng75970D	-2.3007	AT5G66780	2.6254
extracellular ligand-gated ion channel protein	BnaC03g41510D	-1.9540	AT3G20300	0.6353
NCED2 / 9-cis-epoxycarotenoid dioxygenase, ABA biosynthesis	BnaC07g35240D	-2.3266	AT4G18350	-2.3385
dehydrin RAB18, DROUGHT-INDUCED 8; RESPONSIVE TO ABA 18	BnaC02g45160D	-2.4544	AT5G66400	0.2016
RD29B=responsive-to-dessication protein	BnaC02g15280D	-1.8993	AT5G52300	1.8376
protease inhibitor/seed storage/lipid transfer protein (LTP)	BnaC04g45500D	-2.4426	AT2G37870	1.6484
glycine-rich cell wall protein-like protein	BnaA08g08680D	-2.2698	AT4G18280	-0.0821
uncharacterized hypothetical protein	BnaC02g05930D	-1.4018	AT5G15190	-1.4789
NFU1 iron-sulfur cluster protein	BnaC09g48550D	-2.6418	AT5G07330	0.8504
sulfate/thiosulfate import ATP-binding protein	BnaA05g30260D	-2.2998	AT3G07350	0.6845
late embryogenesis abundant protein, LEA 4-5	BnaC09g48810D	-2.4010	AT5G06760	3.2119
stress induced KIN2-like / LEA	BnaC03g32950D	-2.4832	AT3G02480	4.7488
HAI2, Highly ABA induced protein phosphatase 2C 3	BnaA09g49440D	-3.1555	AT1G07430	5.5102
NCED3 / nine-cis-epoxycarotenoid dioxygenase3 / ABA biosynthesis	BnaC01g36910D	-4.4987	AT3G14440	1.2681
Probable desiccation-related protein LEA14	BnaAnng17910D	-2.7124	AT1G01470	1.0282
Chloroplast vesiculation, histone deacetylase-like protein	BnaA04g15010D	-2.0875	AT2G25625	2.6498
late embryogenesis abundant (LEA) domain-containing protein	BnaC05g35990D	-2.9309	AT3G17520	4.0446
WD40 domain-containing protein	BnaA08g02680D	-2.8430	AT1G49450	-1.2911
Phosphate transporter PHO1 / EXS (ERD1/XPR1/SYG1) family protein	BnaC06g30650D	-2.4735	AT1G69480	-2.7081
MAPKKK18	BnaC05g03130D	-2.2738	AT1G05100	1.0401
group 3 late embryogenesis abundant (LEA) protein	BnaC05g37670D	-2.9390	AT3G15670	1.2412
Ninja-family protein AFP3 (ABI five binding protein 3)	BnaA09g02630D	-3.3789	AT3G29575	0.1053
MYB102 myb-related protein	BnaA01g11280D	-4.4374	AT4G21440	1.0416
ABA1, first step in ABA synthesis	BnaA09g07610D	-7.9401	AT5G67030	-0.4620
abscisic acid 8'-hydroxylase 4 / CYP707A4	BnaC05g33590D	-11.253	AT3G19270	1.2412
ethylene-responsive transcription factor ERF53	BnaC08g36140D	-10.269	AT2G20880	-1.1905

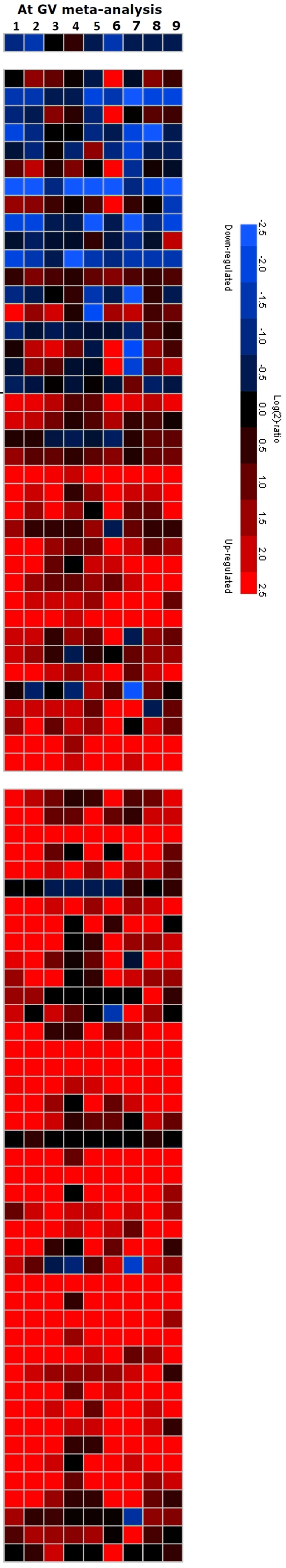

Supplement: Supplementary file 9 — Fig. S9 Differentially expressed genes related to abscisic acid (ABA) signalling as identified by RNA‐Seq of Verticillium longisporum (Vl43)‐infected oilseed rape (Bn) and Arabidopsis (At) roots at 6 days post‐inoculation. The line separates up‐and down‐regulated genes in response to the Vl43 infection of Brassica napus. The meta‐analysis of the orthologous Arabidopsis counterparts in response to various abiotic stress treatments focuses on ABA (1: AT‐00110, 2: AT‐00420), high light (3: AT‐00246), cold (4: AT‐00640), drought (5: AT‐00560, 6: AT‐00419), heat (7: AT‐00387), osmotic (8: AT‐00120) and salt stress (9: AT‐00534) treatments. [file MPP-20-1645-s009.pdf]
