# Supplementary material for: Suppression of abscisic acid biosynthesis at the early infection stage of Verticillium longisporum in oilseed rape (Brassica napus)
Source: Mol Plant Pathol. 2019 Oct 11;20(12):1645–61. doi: 10.1111/mpp.12867 (PMC6859492; doi:10.1111/mpp.12867)
Supplement: Supplementary file 13 — Table S4 Verification of RNA‐Seq data by qPCR (comparison of log2 values), P ≤ 0.05 (*), P ≤ 0.01 (**), P ≤ 0.001 (***). [file MPP-20-1645-s013.docx]

**Tab. S4.** Verification of RNAseq data by qPCR (comparison of log_2_ values)

p-value ≤ 0.05 (*), p ≤ 0.01 (**), p ≤ 0.001 (***)

| **Gene** | **AGI code** | **Bn identifier** | **RNAseq** | **qPCR** |
| --- | --- | --- | --- | --- |
| **Fungal Infection Marker** | | | | |
| Chitinase | AT2G43590 | BnaA09g15440D  BnaC03g19370D | 3.366  0.502 | 1.558*** |
| **SA-associated** | | | | |
| PR1a | AT2G14610 | BnaA03g38630D  BnaC03g45470D | 9.341  4.884 | 8.939*** |
| **SA/JA-associated** | |  |  |  |
| WRKY70 | AT3G56400 | BnaC06g15910D BnaA07g16850D | 2.181  2.571 | 2.803* |
| **SA/ET-associated** | | | | |
| WRKY53 | AT4G23810 | BnaC01g15640D  BnaA01g13440D | 2.229  1.949 | 1.464** |
| bHLH129 | AT2G43140 | BnaC04g02750D  BnaA03g20070D | 2.199  1.833 | 2.277 |
| **ET-associated** | | | | |
| ACO1 | AT2G19590 | BnaC08g36640D  BnaA09g44060D | 0.937  0.518 | 1.039 |
| **JA-associated** | | | | |
| LOX3 | AT1G17420 | BnaC08g48320D  BnaA08g23120D | 4.848  0.000 | 9.009 |
| **ABA-associated up-regulated** | | | | |
| MATE | AT5G52050 | BnaC02g15390D  BnaA03g12860D | 3.469  0.467 | 4.218* |
| PYL6 | AT2G40330 | BnaA04g29300D  BnaC04g47050D | 3.362  3.423 | 2.083 |
| WRKY18 | AT4G31800 | BnaC07g43490D  BnaAnng32610D | 1.078  2.034 | 1.193** |
| **ABA-associated down-regulated** | | | | |
| NCED3 | AT3G14440 | BnaA03g33390D  BnaC01g36910D | -2.212  -2.249 | -1.556*** |
| LEA14 | AT1G01470 | BnaAnng17910D  BnaCnng23520D | -2.712  -2.107 | 0.301 |
| AFP3 | AT3G29575 | BnaA09g02630D  BnaC09g02080D | -3.379  -1.901 | -0.997* |
| Hva22D | AT4G24960 | BnaA03g47030D  BnaC01g16810D | -1.874  -0.393 | -0.748* |
| unknown | AT3G20300 | BnaC03g41510D  BnaA05g18750D | -1.954  -1.196 | -1.135* |
| WRKY57 | AT1G69310 | BnaC06g25390D  BnaA07g24310D | -1.366  -0.782 | -1.006*** |
| MYB102 | AT4G21440 | BnaA01g11280D  BnaC03g64160D | -4.437  -1.717 | -2.160*** |
