# Supplementary material for: Suppression of abscisic acid biosynthesis at the early infection stage of Verticillium longisporum in oilseed rape (Brassica napus)
Source: Mol Plant Pathol. 2019 Oct 11;20(12):1645–61. doi: 10.1111/mpp.12867 (PMC6859492; doi:10.1111/mpp.12867)
Supplement: Supplementary file 14 — Table S5 Brassica napus primers for qPCR. [file MPP-20-1645-s014.docx]

**Tab. S5 *Brassica napus* primer for qPCR**

| **Gene** | **At AGI** | **Bn alleles in RNAseq** | **forward** | **reverse** | **[bp]** | **T [°C]** |
| --- | --- | --- | --- | --- | --- | --- |
| Actin2/7 | AT5G09810 | BnaA10g22340D | TCTACAACGAGCTCCGTGTTG | TGTGAGACACACCATCACCA | 216 | 57 |
| PP2A | AT1G13320 | BnaA06g33370D | CAATGACGATGACGATGAGGTG | ATGCTCAACCAAGTCACTCTCC | 208 | 59 |
| Chitinase | AT2G43590 | BnaA09g15440D, BnaC09g51720D | gcattgtgacacagcagttc | gcttgtatgtagcagaagtg | 202 | 57 |
| PR1 | AT2G14610 | BnaA03g38630D, BnaC03g45470D | TCAGGTTGTTTGGAGAAAGTC | CATTAGTAAGGCTTCTCGTTCACA | 126 | 57 |
| ETR2 | AT3G23150 | BnaA01g24090D, BnaC01g31130D | CACTGCCATTGCACCTAGCTCTT | CAACTCCATTGATCCCAATCTGC | 200 | 59 |
| ACO1 | AT2G19590 | BnaCnng67880D, BnaA09g44060D, … | GAGAAGTTTTACCAGTCAGAGATGG | GGGTATTTAGCCACTTTTGTCC | 296 | 59 |
| LOX3 | AT1G17420 | BnaC08g48320D, BnaC08g37760D, … | GTGAGGAACAAGAACAAGGAG | CGTTTTTGGATCAAGTTCGGTG | 123 | 59 |
| PDF1.2 | AT5G44420 | BnaC02g23400D, BnaA07g32150D, … | cagctcacaagtgtatttgctac | ttacacaaaactaaccaccaaagag | 72 | 59 |
| MATE | AT5G52050 | BnaC02g15390D, BnaA02g11060D | TTCTCCACTTGCCCATCAC | TCGAGACAAACCGATACAC | 247 | 57 |
| PYL6 | AT2G40330 | BnaC03g22610D, BnaA04g29300D, … | TCCGAGTCGTCTCTGGTCTC | CTGTAGTTCTGAAGCCTGTG | 121 | 59 |
| NCED3 | AT3G14440 | BnaA01g29390D, BnaC01g36910D, … | ATGCTATTCTACGCCAGAGC | TGGGCGATCATTGTGGATTC | 212 | 57 |
| LEA14 | AT1G01470 | BnaAnng17910D, BnaCnng23520D | AAAGACGTGAACCGTGACTC | CATCAAGAACCGTCATGTCC | 181 | 57 |
| AFP3 | AT3G29575 | BnaC09g02080D, BnaA09g02630D^1^ | GATATTTGCTCGTTTCTTGGG | TCTCTAGGGATCTCTTCTCCG | 122 | 57 |
| Hva22D | AT4G24960 | BnaA03g47030D, BnaC01g16810D | gagaacagttcaagaaacacgg | GTGAGCCTCGTGTCCCTCC | 107 | 59 |
| Unknown ↓ | AT3G20300 | BnaC03g41510D, BnaC05g31730D, … | CGCTGCTCATTCTACTACGAAG | CGTCTCCATAATCCTCACTG | 216 | 59 |
| MYB102 | AT4G21440 | BnaA01g11280D, BnaC01g41180D, … | TCTTGAACACGCCATCCTC | CGAGATTACTGCAATAGCTTTCC | 113 | 59 |
| bHLH129 | AT2G43140 | BnaA04g24850D, BnaC04g02750D, … | GCTGAAGAAGCTACAAGAAC | GGTGTTGAAGACCTTTGATATGCTC | 101 | 57 |
| WRKY18 | AT4G31800 | BnaC03g67380D, BnaAnng32610D^2^, . | CATAACCACTTAGGTCCAAACG | TATAGCAGCCGCAAGAGCAG | 207 | 59 |
| WRKY33 | AT2G38470 | BnaA03g17820D, BnaC03g21360D, … | TGTGGGAGTGAACCTGAAGC | TCGGCTCTCTCACTGTCTTG | 91 | 59 |
| WRKY53 | AT4G23810 | BnaA01g13440D, BnaC01g15640D^3^ | AGAACTGTTGGGCAACGAAG | AGTGGTGGTGAGTTTAGGTC | 167 | 57 |
| WRKY57 | AT1G69310 | BnaC06g25390D, BnaA07g27840D | GAAGAGAGTGGAACGATCATC | ATTATGAGGGTCGTGAGCTG | 124 | 59 |
| WRKY70 | AT3G56400 | BnaA04g02560D, BnaC08g27340D, … | GGGTAAAAGAGGATGCTAC | CTTGCTTTGTTGCCTTGCAC | 185 | 59 |

Dots indicate more than two existing copies of the respective gene

1. 1 nt longer amplicon
2. 27 nt shorter amplicon
3. 3 nt shorter amplicon
