# Supplementary material for: Suppression of abscisic acid biosynthesis at the early infection stage of Verticillium longisporum in oilseed rape (Brassica napus)
Source: Mol Plant Pathol. 2019 Oct 11;20(12):1645–61. doi: 10.1111/mpp.12867 (PMC6859492; doi:10.1111/mpp.12867)
Supplement: Supplementary file 15 — Table S6 Arabidopsis thaliana primers for qPCR. [file MPP-20-1645-s015.docx]

**Tab. S6 *Arabidopsis thaliana* primer for qPCR**

| **Gene** | **At AGI** | **forward** | **reverse** | **[bp]** | **T [°C]** |
| --- | --- | --- | --- | --- | --- |
| Actin2 | AT3G18780 | accttgctggacgtgaccttactgat | gttgtctcgtggattccagcagctt | 298 | 58 |
| PP2A | AT1G13320 | CAATGACGATGACGATGAGGTG | ATGCTCAACCAAGTCACTCTCC | 208 | 59 |
| NCED3 | AT3G14440 | CGCTTGGGAAGAGCCAGAAA | GAGATGATCGGACGGCGAGT | 162 | 59 |
| WRKY57 | AT1G69310 | ACGGTGAAGAAGAGAGTAGAACG | GTCGTGTGCAGTGAGGATTC | 123 | 59 |
| PR1a | AT2G14610 | TTCTTCCCTCGAAAGCTCAA | AAGGCCCACCAGAGTGTATG | 174 | 58 |
| PDF1.2 | AT5G44420 | CGCTGCTCTTGTTCTCTTTGC | TCCATGTTTGGCTCCTTCAA | 154 | 59 |
| ETR2 | AT3G23150 | cgttggaatttcacaggtcgatgag | cgtcttcgcagttacatcgtgga | 213 | 59 |
