# Supplementary material for: Suppression of abscisic acid biosynthesis at the early infection stage of Verticillium longisporum in oilseed rape (Brassica napus)
Source: Mol Plant Pathol. 2019 Oct 11;20(12):1645–61. doi: 10.1111/mpp.12867 (PMC6859492; doi:10.1111/mpp.12867)
Supplement: Supplementary file 16 — Table S7 Primer used for Arabidopsis T‐DNA genotyping and fungal quantification. [file MPP-20-1645-s016.docx]

**Tab. S7 Primer used for Arabidopsis T-DNA genotyping and fungal quantification**

| **Gene** | **Identifier** | **LP/FW** | **RP/Rev** | **[bp]** | **T [°C]** |
| --- | --- | --- | --- | --- | --- |
| NCED3 | AT3G14440 | ACAGAGGCTCTCCTCCGTAAC | GTCAGCCACGAGAAGCTACAC | 1285 | 61 |
| AAO3 | AT2G27150 | TTCTATTGGAAATGCATTGCC | TAAAACATCGGATGAACCTCG | 1071 | 55 |
| BnPP2A | BnaA06g33370D | CAATGACGATGACGATGAGGTG | ATGCTCAACCAAGTCACTCTCC | 208 | 59 |
| Verticillium ITS | OLG70/71 | CAGCGAAACGCGATATGTAG | GGCTTGTAGGGGGTTTAGA | 261 | 58 |
